# Comparative Adsorption of Phenol and *p*-Chlorophenol on a Chitosan–Cellobiose Dimer in an Aqueous Medium: A DFT Study of Hydrogen Bonding and Noncovalent Interactions

**DOI:** 10.3390/molecules31111871

**Published:** 2026-05-29

**Authors:** Jose Alfonso Prieto Palomo, Juan Jose Carrascal, Joaquín Alejandro Hernández Fernández

**Affiliations:** 1 Chemistry Program, Department of Natural and Exact Sciences, San Pablo Campus, Universidad de Cartagena, Cartagena de Indias D.T. y C., Cartagena 130015, Colombia; 2 Grupo de Investigación GIA, Fundacion Universitaria Tecnologico Comfenalco, Cr 44 D N 30A, 91, Cartagena 130015, Colombia; invest.ambiental@tecnologicocomfenalco.edu.co; 3 Department of Natural and Exact Science, Universidad de la Costa, Barranquilla 080002, Colombia

**Keywords:** adsorption, chitosan, cellobiose, phenol, *p*-chlorophenol, density functional theory, non-covalent interactions

## Abstract

A comparative study was carried out using density functional theory of the adsorption of phenol and *p*-chlorophenol on two molecular models of biopolymers in aqueous medium: a chitosan dimer and cellobiose. Twelve adsorbent–adsorbate complexes with three initial orientations per system were optimized, and their structural, electronic, and non-covalent properties were analyzed using boundary orbitals, molecular electrostatic potential, NCI/RDG, and QTAIM. In all four systems, the most stable geometry corresponded to the anchoring of the contaminant hydroxyl group to an adsorbent hydroxyl group, identifying O–H···O as the guiding motif of molecular recognition. However, conformational selectivity was strongly dependent on the adsorbent and the aromatic substituent. For phenol, the alternative orientations were 2.7 and 21.2 kcal mol^−1^ in chitosan and 6.6 and 48.9 kcal mol^−1^ in cellobiose. For *p*-chlorophenol, chitosan showed a much more severe discrimination, with penalties of 43.6 and 46.44 kcal mol^−1^. In contrast, in cellobiose, the alternative orientations remained close to the minimum, with differences of 5.1 and 3.5 kcal mol^−1^. The effect of Cl was also reflected in the electron topology: PC increased from 3.2 × 10^−2^ to 6.34 × 10^−2^ a.u. in chitosan and from 3.2 × 10^−2^ to 4.2 × 10^−2^ a.u. in cellobiose, while |V|/G went from 3.6 to 7.5 in chitosan and from 3.00 to 3.1 in cellobiose. Overall, the results show that *p*-chlorophenol interacts more intensely and selectively with chitosan, whereas cellobiose favors a more flexible, less topologically differentiated adsorption. These results clarify how a para-chloro substituent reorganizes hydrogen-bond-driven adsorption on two biopolymer microenvironments with different functional heterogeneity.

## 1. Introduction

Phenolic compounds remain a persistent concern in aquatic environments because their toxicity, mobility, and chemical stability enable adverse effects even at low concentrations [1,2]. Phenol and chlorinated phenols are particularly relevant because they are widely used in industrial processes and are frequently found in contaminated effluents [3]. Their removal remains challenging because conventional treatments may be inefficient when these molecules are present at low concentrations, coexist with other organic species, or exhibit high resistance to degradation [4,5]. Adsorption has therefore become one of the most practical strategies for phenolic contaminant mitigation, owing to its operational simplicity, adaptability to different water matrices, and potential for designing selective, regenerable, and environmentally compatible adsorbents [2,4,5].

Biopolymer-based adsorbents have gained increasing attention in this context because they combine structural abundance, low environmental impact, chemical tunability, and a high density of polar functional groups [6,7]. Chitosan is particularly attractive because its structure contains both amino and hydroxyl groups, providing chemically diverse interaction sites that can participate in hydrogen bonding, electrostatic interactions, and donor–acceptor processes [8,9,10]. Cellulose and cellulose-derived fragments represent another relevant family of biopolymeric adsorbents because their hydroxyl-rich surfaces can establish extended hydrogen-bonding networks and contribute to molecular recognition through directional polar contacts [11,12,13,14]. In both cases, adsorption efficiency is not governed only by the number of available functional groups, but also by the accessibility, orientation, polarity, and local electronic environment of the active sites [7,15].

Despite extensive experimental evidence supporting the use of chitosan- and cellulose-based materials for removing organic pollutants, the molecular origin of their selectivity toward structurally related phenolic compounds remains insufficiently resolved [8,11,16]. Most adsorption studies report macroscopic parameters such as removal efficiency, adsorption capacity, or kinetic behavior. Still, these observables do not directly explain how specific substituents reorganize the adsorbate–adsorbent interaction at the atomic scale. This limitation becomes particularly important for phenol and *p*-chlorophenol, where the replacement of a hydrogen atom by chlorine modifies the electronic distribution, dipole response, polarizability, and steric profile of the aromatic adsorbate [16]. Consequently, the adsorption of substituted phenols cannot be rationalized only from the presence of the phenolic hydroxyl group; it requires a molecular description of the competition between hydrogen bonding, electrostatic polarization, dispersion forces, and substituent-induced electronic effects.

The unresolved chemical question is whether chlorine substitution promotes the same recognition pattern on different biopolymer microenvironments or whether the functional diversity of the adsorbent controls the final adsorption geometry. In chitosan, amino and hydroxyl groups can act as competing interaction sites, allowing O–H···O and O–H···N hydrogen-bonding motifs to contribute differently to adsorption stabilization. In contrast, cellobiose provides an exclusively hydroxylated environment that is more representative of cellulose-like recognition sites. Therefore, a direct comparison between these two molecular models can clarify whether *p*-chlorophenol adsorption is mainly controlled by the phenolic OH group or by the combined electronic effect of chlorination and the chemical heterogeneity of the adsorbent.

Computational chemistry provides a suitable route to address this problem because it allows the evaluation of adsorption geometries, interaction energies, charge redistribution, and non-covalent interaction patterns with atomic-level resolution [17]. Density functional theory has been widely used to rationalize adsorption mechanisms in water treatment systems, particularly to identify preferential binding sites, estimate relative interaction strengths, and connect electronic structure to molecular recognition [17]. In addition, reduced molecular models such as chitosan oligomers and cellobiose have been shown to capture the local chemistry of amino- and hydroxyl-containing polysaccharide fragments, making them useful approximations for analyzing specific adsorbent–adsorbate contacts without introducing the complexity of extended polymeric surfaces [18,19].

Accordingly, phenol and *p*-chlorophenol adsorption was analyzed on two representative biopolymer fragments: a chitosan dimer, selected as a minimal model containing both amino and hydroxyl recognition sites, and cellobiose, selected as a cellulose-derived model containing only hydroxyl-rich interaction domains. The central hypothesis is that chlorine substitution does not merely change the adsorption energy of phenol derivatives but also reorganizes the balance among hydrogen bonding, electrostatic polarization, and dispersion, depending on the functional diversity of the adsorbent. By comparing adsorption orientations, relative stabilities, interatomic distances, electrostatic potential distributions, atomic charges, and topological descriptors of non-covalent interactions, the analysis establishes how phenolic chlorination modifies molecular recognition in aqueous biopolymer microenvironments.

This molecular-level comparison contributes to the chemistry of biopolymer-based adsorption by identifying which interaction motifs govern the stabilization of phenol and *p*-chlorophenol on hydroxyl- and amino-containing adsorbents. The resulting interpretation provides a mechanistic basis for understanding why chitosan- and cellulose-derived materials may display different selectivity toward chlorinated phenolic contaminants, offering information that cannot be obtained directly from adsorption capacity data alone.

## 2. Results and Discussion

### 2.1. Conformation of the Model Adsorbents

**Figure 1:**
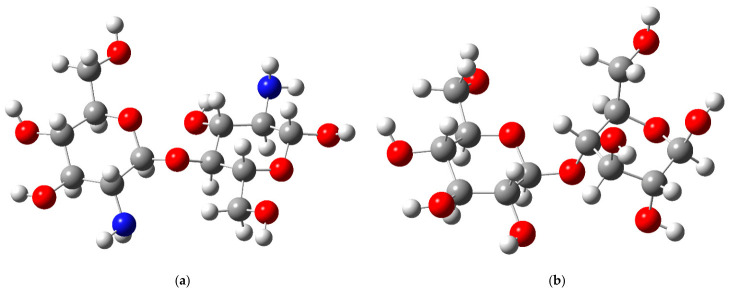
Optimized geometries of the model adsorbents used in this study: (**a**) chitosan dimer and (**b**) cellobiose. Carbon, oxygen, nitrogen, and hydrogen atoms are depicted in gray, red, blue, and white, respectively.

The optimized geometries of chitosan dimer and cellobiose revealed that both fragments retain sufficient structural flexibility to modify the accessibility of their functional groups and, consequently, the chemical microenvironment available for adsorption (Figure 1). In the case of the chitosan dimer, the minimum energy conformation exhibited a spatial arrangement that was not fully extended, with a slight curvature of the saccharide skeleton and a differentiated distribution of hydroxyl and amino groups along the structure. This organization is particularly relevant, since it generates a heterogeneous environment that can establish localized interactions through hydrogen bonds and, at the same time, favor cooperative recognition among multiple functional sites of the adsorbent. From a chemical perspective, this characteristic is consistent with the adsorptive behavior widely attributed to chitosan, whose efficacy is often attributed to the coexistence of –OH and –NH2 centers within the same structural unit.

On the other hand, cellobiose presented a topology dominated exclusively by hydroxyl groups oriented in complementary directions around the β-(1 → 4)-glycosidic motif. Unlike the chitosan dimer, the absence of amino groups gives this system a more uniform, markedly polar adsorbent surface, in which molecular recognition depends mainly on the relative arrangement of the –OH groups and on the possibility of forming O–H···O hydrogen bonds. In this sense, cellobiose constitutes an appropriate molecular model for representing the local behavior of cellulose in adsorptive processes governed by polar interactions and the spatial organization of hydroxyls.

From an electronic point of view, the two adsorbents exhibited high overall stability, though with subtle differences in their electronic response (Table 1 and Table S1). The chitosan dimer had a HOMO energy of −8.9 eV and a LUMO energy of 3.1 eV, which corresponds to a HOMO–LUMO separation of 12.0 eV. In contrast, cellobiose showed HOMO and LUMO values of −9.4 and 3.0 eV, respectively, with an energy gap of 12.4 eV. The slightly greater HOMO–LUMO separation observed for cellobiose suggests a more rigid electronic response, consistent with a chemically more homogeneous structure and dominated by hydroxyl groups. On the other hand, the smaller gap of the chitosan dimer suggests a greater capacity for local electron reorganization, attributable to the simultaneous presence of amino and hydroxyl groups.

In comparative terms, these results indicate that both models capture two distinct scenarios of molecular recognition. While chitosan dimer defines a more versatile and functionally diverse environment, cellobiose represents a more uniform surface, where adsorption is governed almost exclusively by hydroxyl–hydroxyl interactions and by geometric adjustments of the adsorbate. This structural and electronic difference forms the basis of the subsequent comparative analysis, as it will allow us to assess whether the adsorption of phenol and *p*-chlorophenol depends mainly on the presence of an additional amino group or whether a properly oriented network of hydroxyl groups is sufficient to stabilize adsorbent–pollutant complexes in aqueous medium.

### 2.2. Relative Stability of Adsorbent–Pollutant Complexes

The stability of the twelve optimized complexes was initially analyzed in terms of relative Gibbs free energy within each adsorbent–pollutant family, to directly compare the orientations generated for the same system without mixing species of different stoichiometry. Under this criterion, the optimization showed a clear and consistent pattern: across the four series studied, orientation 1, corresponding to the initial anchoring of the pollutant’s hydroxyl group to a hydroxyl group of the adsorbent, yielded the lowest-energy minimum. This result indicates that, in both chitosan dimer and cellobiose, intermolecular recognition is mainly governed by O–H···O interactions. In contrast, alternative orientations are only stabilized secondarily or locally. Table 2 summarizes the relative Gibbs free energies of each complex, as well as the shortest interfragment polar distances obtained after optimization.

In chitosan dimer-based systems, the preference for orientation 1 was especially marked. For the chitosan–phenol complex, orientation 1 was found to be the most stable. In contrast, orientation 2, in which the hydroxyl group of the contaminant is initially directed towards the amino group, was 2.7 kcal mol^−1^ above the minimum. The mixed orientation was considerably less favorable, with a penalty of 21.2 kcal mol^−1^. This behavior indicates that although the chitosan amino group may be involved in adsorbate stabilization, anchoring through a hydroxyl–hydroxyl interaction remains the preferred mode of recognition for phenol. In the minimal complex, the shortest polar distances were 2.5 Å and 2.4 Å, consistent with a coupling dominated by O–H···O.

**Figure 2:**
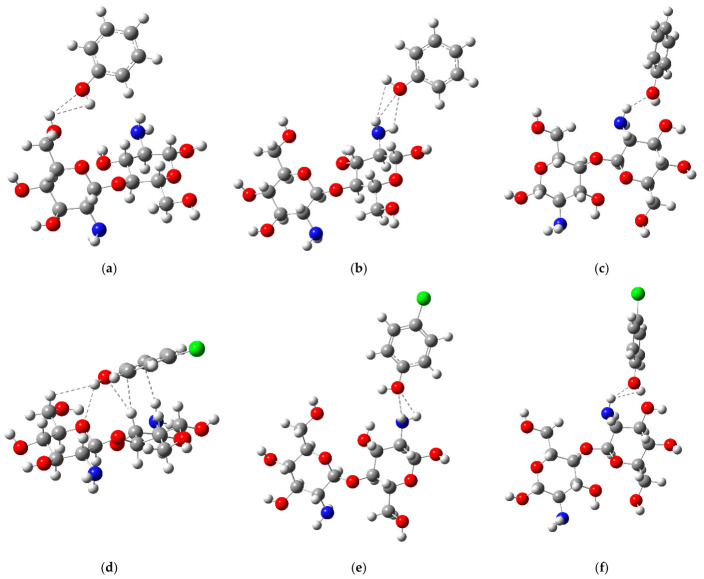
Optimized chitosan dimer complexes with (**a**–**c**) phenol and (**d**–**f**) *p*-chlorophenol. In each series, the three orientations evaluated are shown: (**a**,**d**) orientation 1, corresponding to the initial anchoring of the hydroxyl group of the pollutant to a hydroxyl group of the dimer; (**b**,**e**) orientation 2, with the hydroxyl group of the contaminant initially directed towards the amino group; and (**c**,**f**) orientation 3, corresponding to the mixed arrangement between two functional groups of the adsorbent.

The case of *p*-chlorophenol over chitosan further reinforces this trend. Orientation 1 remained at the global minimum, while orientations 2 and 3 were destabilized at 43.6 and 46.4 kcal mol^−1^, respectively (Figure 2). This much larger energy difference suggests that the presence of the chlorine atom restricts the thermodynamic viability of alternative geometries and more decisively favors the initial coupling of the pollutant’s hydroxyl group with the adsorbent’s hydroxyl groups. In the more stable complex, the two shortest polar interfragment distances were practically equivalent (2.5 and 2.5 Å), indicating a more symmetrical and well-defined adsorption motif than that observed for the phenol system. These results show that chitosan does not necessarily exploit its amino group as a preferential site of recognition against these phenols, but preferentially stabilizes complexes in which anchoring predominates over its network of hydroxyl groups.

For cellobiose-based complexes, orientation 1 was also the most stable in both series, though with a somewhat different energy landscape. In the cellobiose-phenol system, orientation 2, corresponding to an arrangement between two hydroxyl groups of the fragment, was 6.6 kcal mol^−1^ above the minimum. In contrast, the semiparallel orientation was highly unfavorable, with a difference of 48.9 kcal mol^−1^. This indicates that, for phenol, cellobiose clearly favors directional recognition governed by the hydroxyl group of the adsorbate and does not readily tolerate a more diffuse aromatic-ring arrangement on the polar surface of the model. At the global minimum, the shortest polar distances were 2.6 Å and 2.8 Å, compatible with an interaction dominated by a single hydroxyl–hydroxyl anchor.

**Figure 3:**
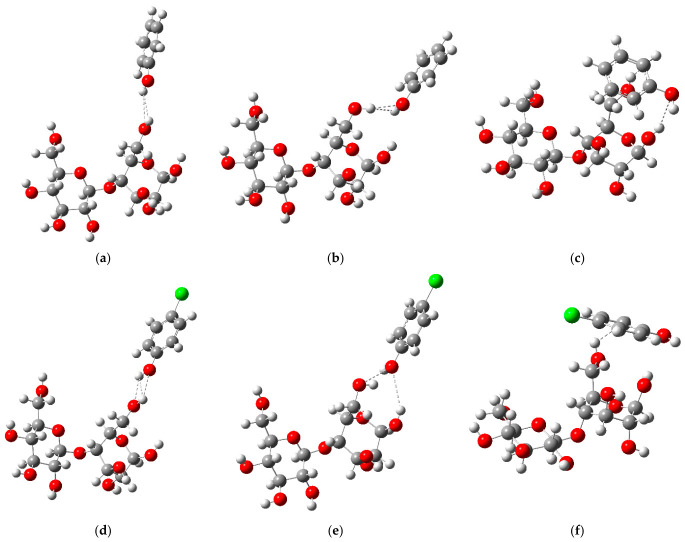
Optimized cellobose complexes with (**a**–**c**) phenol and (**d**–**f**) *p*-chlorophenol. In each series, the three orientations evaluated are shown: (**a**,**d**) orientation 1, corresponding to the anchoring of the hydroxyl group of the contaminant towards a donor/acceptor hydroxyl group of the cellobiose; (**b**,**e**) orientation 2, located between two hydroxyl groups of the fragment; and (**c**,**f**) orientation 3, corresponding to the semiparallel arrangement of the aromatic ring near the polar surface.

The behavior of *p*-chlorophenol toward cellobiose, however, showed a differentiating characteristic (Figure 3). Although orientation 1 remained the most stable, orientations 3 and 2 were only 3.5 and 5.1 kcal mol^−1^ above the minimum, respectively. This pattern suggests a less restrictive conformational landscape than that observed for phenol, implying that the incorporation of the chlorine atom increases the system’s tolerance to geometries not strictly directed by the main hydrogen bond. In the minimal complex, the shortest polar contacts were reduced to 2.2 and 2.3 Å, shorter values than those observed for phenol–cellobiose, indicating a more compact anchorage. However, the energetic closeness of the semiparallel arrangement reveals that *p*-chlorophenol can be stabilized not only by hydroxyl–hydroxyl coupling, but also through a greater contribution of extended non-covalent contacts.

From a global perspective, the relative stability results allow us to establish three central observations. First, across the four systems studied, the hydroxyl group of the pollutant served as the dominant structural anchor, confirming that hydrogen-bond formation with the adsorbent surface is the guiding factor of the adsorption process. Second, the chitosan amino group did not emerge as the preferred site of initial recognition, since even in systems where it was explicitly oriented towards the contaminant, it did not lead to a thermodynamic minimum. Third, the comparison between phenol and *p*-chlorophenol suggests that chlorine substitution does not eliminate the hydroxyl group’s central role. Still, it does modify the energetic flexibility of the adsorptive landscape. In chitosan, it reinforces the preference for a well-defined hydroxyl-based geometry, while in cellobiose, it allows less directional arrangements to become relatively more competitive.

Consequently, the relative stability of the complexes indicates that molecular recognition in these biopolymers depends not only on the presence of active functional groups but also on how the contaminant’s electronic structure modulates the accessibility and relative weight of alternative interactions. This observation forms the basis of the subsequent analysis on the specific effect of the chlorine substituent on the architecture of the adsorbent–adsorbate complex.

To complement the optimized structures shown in Figure 2 and Figure 3 without overloading the molecular representations, the key interfragment polar distances are summarized in Table 3. These distances provide a quantitative description of the dominant hydrogen-bonding and polar-contact motifs associated with each optimized adsorption geometry.

### 2.3. Effect of the Chlorine Substituent on Molecular Recognition

The comparison between phenol and *p*-chlorophenol constitutes the conceptual core of this study, since both molecules retain the same phenolic hydroxyl group, but differ in the presence of a chlorine atom at the para position. From the structural and electronic point of view, this modification can influence in two complementary ways: on the one hand, the –OH group can continue to act as the dominant center of intermolecular recognition; on the other hand, the halogenated substituent can alter the electronic distribution of the ring, its polarizability, and the way in which the adsorbate is arranged on the surface of the biopolymer. Consequently, the effect of Cl should not be analyzed only in terms of “greater” or “lesser” adsorption, but as a factor that reorganizes the architecture of the complex and the relative hierarchy of accessible orientations.

The results obtained indicate that, in both chitosan and cellobiose, the incorporation of the chlorine atom does not modify the primary mode of recognition, since, in both adsorbents, the most favorable orientation remains that in which the hydroxyl group of the contaminant is directed towards a hydroxyl group of the adsorbent. This result confirms that the phenolic –OH group remains the directing center of the adsorptive process, even after substitution in the para position. However, the presence of Cl significantly altered the energetic landscape of the alternative orientations, indicating that its effect is not neutral with respect to molecular recognition.

In the chitosan dimer-based system, the presence of the chlorine substituent led to a marked difference between the global minimum and the alternative configurations. While for phenol the orientation initially directed towards the amino group of chitosan remained relatively close to the minimum, with a penalty of only 2.7 kcal mol^−1^, in the system with *p*-chlorophenol that same orientation was much more destabilized, with a difference of 43.6 kcal mol^−1^. The mixed orientation followed the same pattern, ranging from 21.2 kcal mol^−1^ for phenol to 46.4 kcal mol^−1^ for *p*-chlorophenol. This behavior suggests that the introduction of the chlorine atom significantly restricts the viability of alternative arrangements on the chitosan surface and favors a more geometrically defined adsorption. In other words, in the presence of the halogenated substituent, the system seems to discriminate more severely between favorable and unfavorable orientations, reinforcing the preference for a specific anchor governed by hydroxyl–hydroxyl interactions.

From a geometric perspective, this trend is accompanied by a more symmetrical pattern in the polar contacts of the minimum complex. In chitosan–phenol–1, distances of 2.6 and 2.4 Å were observed, while in chitosan–p–chlorophenol–1, both interactions converged to practically equivalent values (2.5 and 2.5 Å). Although the difference is subtle, this symmetry suggests a more balanced arrangement of *p*-chlorophenol over the hydroxylated environment of the adsorbent. Therefore, the effect of Cl on chitosan is not so much manifested in a change in the main anchorage site as in a stiffening of the adsorptive landscape, where the global minimum is better defined relative to alternative configurations.

The behavior on cellobiose was different and, from a mechanistic point of view, more revealing. In this system, orientation 1 also remained the most stable for both contaminants, thereby reconfirming the dominant role of the adsorbate hydroxyl group as the recognition center. However, the substitution with chlorine modified the energy proximity of the other orientations. For phenol, the semiparallel orientation was clearly unfavorable, with a difference of 48.9 kcal mol^−1^ with respect to the minimum. On the other hand, for *p*-chlorophenol, the same arrangement was only 3.5 kcal mol^−1^ above the most stable orientation, representing a drastic reduction in the energy penalty. The orientation between two hydroxyl groups also became relatively different and, from a mechanistic point of view, more revealing, with a difference of 5.1 kcal mol^−1^. This result indicates that, in cellobiose, the presence of the chlorine atom widens the accessible conformational space and allows arrangements less strictly directed by a single hydrogen bond to remain thermodynamically close to the global minimum.

Comparing polar distances in the minimum cellobiose complexes reinforces this reading. The cellobiose–phenol–1 complex had contacts of 2.6 and 2.8 Å, while in cellobiose–p–chlorophenol–1, these contacts were shortened to 2.2 and 2.3 Å. This contraction suggests a more compact main interaction in the chlorinated derivative. However, the fact that orientations 2 and 3 remain relatively close in energy indicates that the stabilization of *p*-chlorophenol does not depend exclusively on a single geometric motif, but can be distributed between a stronger polar anchor and a greater contribution of extended non-covalent contacts associated with the substituted ring. Thus, in cellobiose, the effect of Cl does not stiffen the adsorption process, as in chitosan, but introduces greater tolerance towards alternative arrangements.

The results show that the effect of the chlorine substituent is dependent on the chemical microenvironment of the adsorbent. Over chitosan, Cl accentuates conformational selectivity and concentrates stabilization within a well-defined hydroxyl-group-based geometry. On the other hand, the same substituent favors a more flexible adsorptive landscape, in which configurations that are not strictly equivalent to the minimum retain relative competitiveness. This contrast indicates that the chlorine atom does not act simply as a passive polarizability modifier, but as an element that redistributes the balance between directionality of the hydrogen bond and more widespread non-covalent contributions, depending on whether the adsorbent surface offers a heterogeneous environment with amino and hydroxyl groups or an exclusively hydroxylated lattice.

Therefore, the main effect of the Cl in position para was not to displace the –OH group as the directing center of recognition, but to modulate the way in which the aromatic ring is arranged and how the orientations accessible in each biopolymer are hierarchical. This observation is key to interpreting the adsorption of substituted phenols in biopolymeric materials, since it shows that small structural modifications in the contaminant may not alter the dominant interaction type, but they may substantially modify the energetics and geometric topology of the adsorptive process.

The structures in which the phenolic hydroxyl group was initially directed toward the amino site of the chitosan dimer were explicitly included as orientation 2.

### 2.4. Electrostatic Potential and Frontier Orbital Analysis

The analysis of the molecular electrostatic potential (MEP) of the isolated adsorbents and adsorbates allowed the identification of the regions of greatest electrostatic predisposition for intermolecular recognition and, consequently, the rationalization of the relative stability observed in the optimized complexes. In general terms, the MEP surfaces showed a heterogeneous distribution of charge over all the systems studied, although with important differences associated with both the nature of the biopolymer and the effect of the halogenated substituent on the contaminant. These differences are particularly relevant because, in adsorptive processes dominated by weak interactions, adsorption does not depend exclusively on the geometric proximity between the fragments, but also on the electrostatic complementarity of their surfaces.

**Figure 4:**
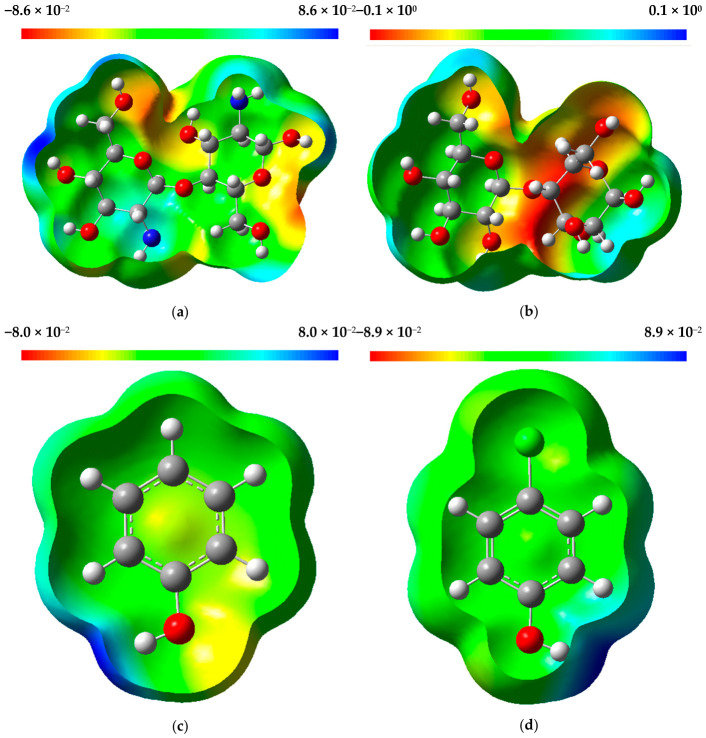
Molecular electrostatic potential surfaces of the isolated species used in this study: (**a**) chitosan dimer, (**b**) cellobiose, (**c**) phenol, and (**d**) *p*-chlorophenol. The extremes of each scale correspond to the regions of most negative and most positive potential, respectively. Values are expressed in atomic units.

In the case of chitosan dimer, the electrostatic potential surface showed a clearly anisotropic distribution, with differentiated electrostatic domains associated with the coexistence of amino and hydroxyl groups (Figure 4a). The amplitude of the surface potential, ranging from −8.6 × 10^−2^ and 8.6 × 10^−2^ a.u., reveals an appreciable polarization of the molecular surface. This heterogeneity is consistent with the chemical nature of chitosan, in which the simultaneous presence of electronegative atoms and potentially donating hydrogens generates multiple regions capable of participating in electrostatic and hydrogen-bonding interactions. From this perspective, chitosan should not be interpreted as a uniformly polar surface but as a functionally diverse environment suitable for stabilizing adsorbates through different modes of local coupling.

Cellobiose also had a polar surface, but with a more homogeneous distribution, dominated by contributions from its hydroxyl groups and glycosidic oxygen (Figure 4b). In this system, the range of electrostatic potential was even wider, from −1.0 × 10^−1^ to 1.0 × 10^−1^ a.u., indicating a marked differentiation between electropositive and electronegative regions. However, unlike chitosan, this polarization is distributed over a less chemically diverse surface, since electrostatic recognition in cellobiose depends almost exclusively on the organization of its –OH groups. Therefore, although cellobiose has a high surface polarity, its interaction capacity is more restricted to hydroxyl–hydroxyl recognition patterns and geometric adjustments of the adsorbate on the fragment.

The isolated adsorbates showed simpler but equally informative electrostatic behavior. In phenol (Figure 4c), the surface area of MEP was mainly determined by the presence of the hydroxyl group, which introduces a clear differentiation between the region associated with phenolic oxygen and the hydrocarbon portion of the ring. The potential range, between −8.0 × 10^−2^ and 8.0 × 10^−2^ a.u., confirms that the highest electron anisotropy in the system is concentrated around the –OH group. At the same time, the rest of the aromatic ring maintains a more uniform distribution. This electrostatic pattern is consistent with the fact that, in the most stable, optimized orientations, the hydroxyl group acts as a directing center for molecular recognition and as the main anchor point to the chitosan and cellobiose surfaces.

For *p*-chlorophenol (Figure 4d), the electrostatic surface retained the dominant role of the hydroxyl group as a key region of interaction, but showed a more widespread redistribution of potential over the aromatic ring as a consequence of the introduction of the chlorine atom. In this case, the MEP range was −8.9 × 10^−2^ to 8.9 × 10^−2^ a.u., slightly higher than that of phenol, suggesting greater electrostatic anisotropy and additional perturbation of the system’s electron density. This result is chemically consistent with the effect of the halogenated substituent, which does not replace the –OH group as the primary site of interaction, but does modify the distribution of the potential over the ring and, therefore, the way in which the adsorbate can be oriented and stabilized on the surface of the biopolymer.

The joint comparison of MEPs allows us to establish an initial mechanistic interpretation of the adsorptive process. On the one hand, the two biopolymers exhibit surfaces sufficiently polarized to stabilize phenolic adsorbates through electrostatic complementarity and hydrogen-bond formation. On the other hand, adsorbates differ in the degree of electronic disturbance associated with aromatic substitution. In particular, the fact that *p*-chlorophenol exhibits a slightly larger potential range than phenol suggests a more anisotropic electrostatic surface, which helps explain why this compound showed different behavior in the adsorption conformational landscape, especially over cellobiose, where less narrowly targeted orientations remained energetically competitive.

The electrostatic discussion is reinforced by the boundary orbitals obtained for the isolated species. Phenol had HOMO and LUMO energies of −8.3 and 1.5 eV, respectively, with a HOMO–LUMO gap of 9.8 eV, while *p*-chlorophenol showed values of −8.2 and 1.3 eV, with a separation of 9.5 eV. The decrease in the energy gap in the chlorinated derivative indicates a somewhat more electronically polarizable structure and greater ease of redistributing electron density in the presence of an adsorbent environment. In contrast, adsorbents had significantly larger gaps: 12.0 eV for chitosan dimer and 12.4 eV for cellobiose. This suggests that, within the adsorbent–adsorbate pair, it is the contaminants that have a greater intrinsic predisposition to undergo electronic reorganization during complex formation.

From this perspective, charge transfer in these systems should be interpreted primarily in qualitative terms as a local electron redistribution induced by the interaction between complementary surfaces, rather than as a process of intense net electron transfer. The combination of polarized MEP surfaces in the adsorbents with HOMO–LUMO gap smaller contaminants supports the idea that adsorption is favored by mutual polarization and weak charge transfer, yet is sufficient to stabilize complexes governed by hydrogen bonds and non-covalent contacts. In this context, *p*-chlorophenol seems to have a greater predisposition to such electronic redistribution than phenol, not because it changes the recognition site of recognition, but because the chlorine atom modifies the electrostatic anisotropy of the ring and facilitates a more flexible electronic response to the adsorbent environment.

The electrostatic potential analysis shows that the initial recognition between adsorbent and adsorbate is strongly conditioned by the complementarity between polar regions and by the ability of the phenolic hydroxyl group to act as a directing center of the interaction. At the same time, the differences between phenol and *p*-chlorophenol indicate that chlorine substitution does not alter the basic electrostatic mechanism of adsorption. Still, it modulates charge redistribution and the system’s electronic architecture, providing a rational basis for the observed energy differences between complexes and orientations.

HOMO and LUMO energies were used here only in a qualitative comparative sense to discuss relative electronic response and orbital redistribution, rather than as direct quantitative estimates of ionization energies or electron affinities.

### 2.5. Non-Covalent Nature of the Adsorption

**Figure 5:**
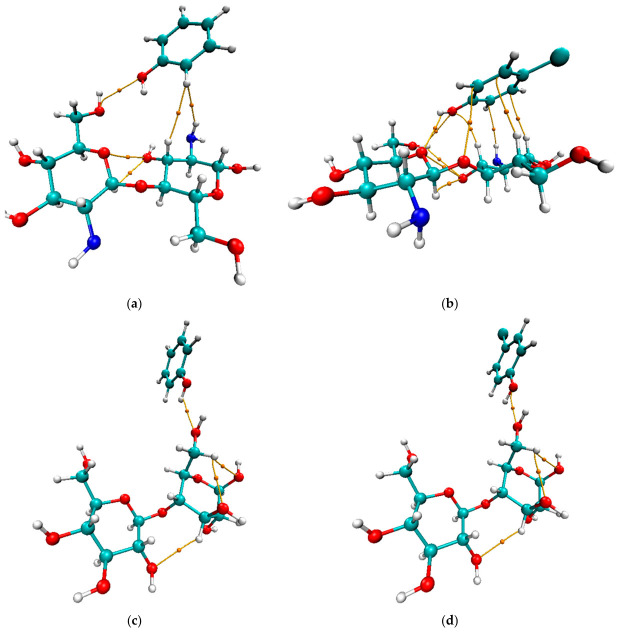
Optimized geometries and intermolecular contact scheme for the representative complexes: (**a**) chitosan–phenol with the hydroxyl group of phenol oriented towards a hydroxyl group of the dimer, (**b**) chitosan–*p*-chlorophenol with the hydroxyl group of the contaminant oriented towards a hydroxyl group of the dimer, (**c**) cellobiose–phenol with the hydroxyl group of phenol directed towards a donor/acceptor hydroxyl group of the cellobiose, and (**d**) cellobiose–*p*-chlorophenol in the equivalent guidance on cellobiose.

**Figure 6:**
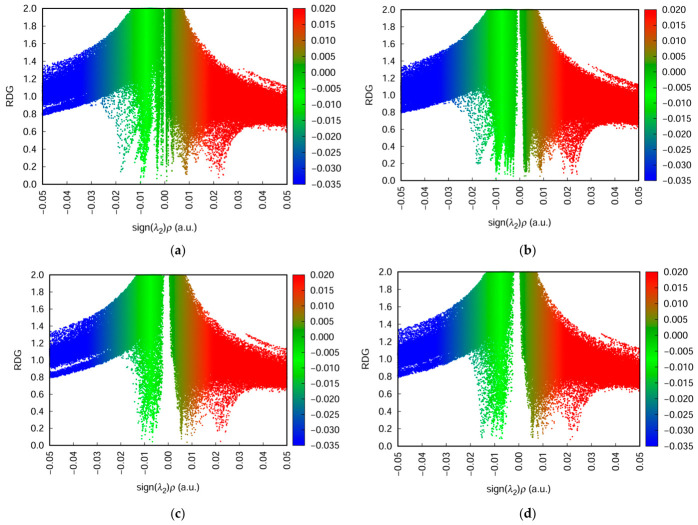
RDG vs sign(λ_2_)p diagrams for the representative complexes: (**a**) chitosan–phenol, with the hydroxyl group of phenol directed towards a hydroxyl group of the dimer; (**b**) chitosan–*p*-chlorophenol, with the hydroxyl group of the contaminant oriented towards a hydroxyl group of the dimer; (**c**) cellobiose–phenol, with the hydroxyl group of phenol oriented towards a hydroxyl donor/acceptor group of cellobiose; and (**d**) cellobiose–*p*-chlorophenol, in equivalent guidance on cellobiose.

To characterize the nature of the interactions responsible for the stabilization of the adsorbent–pollutant complexes, the four most representative systems of the study were selected: chitosan–phenol in the orientation in which the hydroxyl group of phenol is directed towards a hydroxyl group of the dimer, chitosan–*p*-chlorophenol in the orientation in which the hydroxyl group of *p*-chlorophenol is oriented towards a hydroxyl group of the dimer, cellobiose–phenol in the orientation in which the hydroxyl group of phenol interacts with a donor/acceptor hydroxyl group of cellobiose, and cellobiose–*p*-chlorophenol in the analogous orientation on cellobiose. The optimized geometries and associated intermolecular contact network are shown in Figure 5, while the RDG vs. sign(λ_2_)p diagrams are presented in Figure 6. The topological parameters extracted at the critical binding point are summarized in Table 4.

Visual inspection of the optimized geometries reveals that the four complexes share a common structural feature: adsorption begins with a directional anchoring of the contaminant’s hydroxyl group to a polar region of the adsorbent. This confirms that the hydrogen bond acts as the guiding motif of molecular recognition. However, the pattern of secondary contacts differs significantly between systems. In chitosan–phenol, stabilization is concentrated in a relatively localized network of polar interactions, in which the hydroxyl group of phenol functions as an anchoring center on the heterogeneous environment of the dimer. In chitosan–*p*-chlorophenol, on the other hand, a denser and more extended network of contacts is observed around the interaction zone, suggesting that the presence of the chlorine atom favors a more complex reorganization of the intermolecular coupling. In cellobiose complexes, adsorption exhibits a more directional and less diverse character, especially in the phenol system. At the same time, the chlorinated derivative again exhibits a broader contact architecture than its unsubstituted analog.

RDG diagrams versus sign(λ_2_)p reinforce this structural interpretation. In the four systems, a well-defined region is distinguished in the negative-sign (λ2)p domain, characteristic of non-covalent attractive interactions, attributable mainly to hydrogen bonds and electrostatically stabilizing contacts. Also, the presence of a large distribution of points around the sign(λ_2_)p values close to zero indicates the simultaneous contribution of weak dispersive or van der Waals interactions, particularly in areas where the aromatic ring approaches the adsorbent surface. Finally, the branch displaced towards positive values corresponds to regions of steric repulsion, expected in compact complexes in which several fragments are spatially close.

Among the systems analyzed, the chitosan–*p*-chlorophenol complex exhibits the strongest non-covalent signature. In the RDG graph, this system exhibits an extended attractive region towards negative values of sign(λ2)p, suggesting a more pronounced stabilizing contribution than in the other complexes. This observation is consistent with the topological parameters obtained by QTAIM, since this system exhibits the highest electron density at the critical binding point (PC = 6.3 × 10^−2^ a.u.) and the highest ∇^2^PC (2.4 × 10^−1^ a.u.). Similarly, the quotient |V|/G reaches 7.5, significantly higher than in the other systems. These results indicate that the interaction in this complex is not only more intense but also more efficient in terms of local electron-density concentration and potential stabilization in the contact region.

The cellobiose–*p*-chlorophenol complex also shows a clear intensification of the interaction relative to its phenol analog. Although the contact network observed in the optimized geometry is less complex than in chitosan, the QTAIM parameters reveal an appreciable increase in electron density at the critical binding point, from 3.2 × 10^−2^ a.u. in cellobiose-phenol to 4.2 × 10^−2^ a.u. in the *p*-chlorophenol system. The simultaneous increase of ∇^2^PC, V, and G confirms that the introduction of the chlorine atom strengthens the attractiveness of the interaction even in an exclusively hydroxylated adsorbent. However, the value of |V|/G remains close to 3.1, only slightly higher than that of the phenol system, suggesting that the effect of Cl on cellobiose translates more into a moderate intensification of the main contact than a radical reorganization of the topological nature of the interaction.

Phenol complexes exhibit simpler and more uniform behavior. In both chitosan and cellobiose, the values of CP are practically equivalent, 3.2 × 10^−2^ and 3.2 × 10^−2^ a.u., respectively, indicating that the primary interaction established by the hydroxyl group of phenol has a very similar intensity in both adsorbents. The differences between these systems are most clearly manifested in the spatial organization of secondary contacts and in the functional environment in which anchoring occurs. In chitosan, the presence of the amino group and a more heterogeneous surface favors a locally richer network of interactions. In contrast, in cellobiose, the recognition retains a cleaner character and is more strictly directed by hydroxyl-hydroxyl interactions. However, the positive values of ∇^2^PC and H, together with negative V and positive G, confirm in both cases that these interactions are attractive, non-covalent, dominated by electrostatic polarization and hydrogen bonding rather than by intense electron sharing.

In global terms, the joint analysis of the geometries, the RDG diagrams, and the QTAIM descriptors allows us to establish the following qualitative order of non-covalent intensity:

Chitosan–*p*-chlorophenol > Cellobiose–*p*-chlorophenol > Chitosan–phenol ≈ Cellobiose–phenol

This order shows that chlorine substitution systematically intensifies the adsorbent–adsorbate interaction, regardless of the biopolymer considered, although the effect is particularly pronounced for chitosan. Therefore, the chlorine atom not only modifies the adsorption energy landscape, as discussed in the previous section, but also alters the non-covalent architecture of the complex, promoting a more intense and extended interaction network. This result is consistent with the higher electrostatic anisotropy and lower HOMO–LUMO gap previously observed for *p*-chlorophenol, and confirms that aromatic substitution effectively modulates the microscopic nature of the adsorptive process.

Consequently, the adsorption of these contaminants on chitosan and cellobiose should not be interpreted as a process governed by a single isolated hydrogen bond, but rather as the result of cooperation between a primary polar anchor and a variable set of secondary dispersive and electrostatic contributions. The relative magnitude of this cooperation depends on both the adsorbent and the substituent on the contaminant, with the chitosan–*p*-chlorophenol system being the clearest example of non-covalent adsorption enhanced by electronic reorganization of the aromatic ring.

### 2.6. Comparative Implications of the Chitosan–Cellobiose System

The comparative analysis between chitosan dimer and cellobiose shows that both adsorbents share the same primary recognition motif, but differ significantly in conformational selectivity, in the electron response of the adsorbent microenvironment, and in the topological intensity of the interaction. In the four systems studied, the most stable orientation corresponded to the initial anchoring of the contaminant hydroxyl group to a hydroxyl group of the adsorbent. However, the way each biopolymer penalizes alternative geometries differs. For phenol, the orientation directed to the second functional environment was located at 2.7 kcal mol^−1^ from the minimum in chitosan, while in cellobiose, the cooperative arrangement between two hydroxyl groups was at 6.6 kcal mol^−1^. The less favorable orientation imposed a penalty of 21.2 kcal mol^−1^ for chitosan and 48.9 kcal mol^−1^ for cellobiose. These values indicate that, compared to phenol, cellobiose discriminates more severely against configurations far from the optimal hydrogen-bonding motif. In contrast, chitosan tolerates secondary orientations more readily that are moderately close to the minimum.

The most marked difference is observed in the *p*-chlorophenol systems. In chitosan, the alternative orientations are strongly destabilized with respect to the minimum, with differences of 43.6 and 46.4 kcal mol^−1^. In contrast, in cellobiose, those same orientations remain much closer, with penalties of only 5.1 and 3.5 kcal mol^−1^. In practical terms, this means that chitosan imposes on *p*-chlorophenol a much more restricted adsorptive landscape, dominated by a single clearly preferred geometry. At the same time, cellobiose allows for greater structural flexibility and keeps alternative configurations accessible without a high energy cost. Therefore, chitosan not only acts as a polar surface, but as a more conformationally selective adsorbent when the adsorbate incorporates a halogenated substituent.

From a geometric point of view, the minimum complexes also show relevant differences. In chitosan–phenol, the two shortest polar distances were 2.5 and 2.4 Å, while in chitosan–p–chlorophenol, both converged to 2.5 Å, suggesting a more balanced and symmetrical interaction in the chlorinated derivative. In cellobiose–phenol, the corresponding distances were 2.6 and 2.8 Å, while in cellobiose–p–chlorophenol, they were shortened to 2.2 and 2.3 Å. This result indicates that, although cellobiose does not impose as marked conformational selectivity as chitosan, it can establish a very compact local anchorage with *p*-chlorophenol. In other words, cellobiose favors a shorter primary contact with the chlorinated derivative, but does not necessarily translate this compaction into greater discrimination between possible orientations.

The electronic properties of isolated adsorbents also help to rationalize this behavior. Chitosan dimer showed a HOMO–LUMO gap of 12.0 eV, while cellobiose showed a slightly greater separation of 12.4 eV. This difference of 0.4 eV indicates that chitosan has a somewhat more flexible electronic response than cellobiose. Although this variation is not extreme, it is consistent with a more heterogeneous adsorbent environment and with a greater capacity for local reorganization. Consequently, chitosan not only offers different functional groups but also a more electronically adaptable surface to accommodate adsorbate-induced disturbances.

Topological comparison reinforces this conclusion. For phenol complexes, the electron density values at the critical binding point were practically equivalent: 3.2 × 10^−2^ a.u. in chitosan and 3.2 ×10^−2^ a.u. in cellobiose. This indicates that, when the adsorbate lacks additional substituents, both biopolymers can stabilize the main contact with comparable intensity. However, the situation changes markedly in the presence of *p*-chlorophenol. In chitosan, CP increased from 3.2 × 10^−2^ to 6.3 × 10^−2^ a.u., i.e., an increase of nearly 99%. In cellobiose, the increase was more moderate, from 3.2 × 10^−2^ to 4.3 × 10^−2^ a.u., equivalent to about 32%. This difference shows that the chlorine substituent intensifies the interaction in both adsorbents, but does so much more effectively in the more functionally diverse environment of chitosan.

The same pattern is observed in the Laplacian electron density. In chitosan, ∇^2^PC increased from 1.4 × 10^−1^ to 2.4 × 10^−1^ a.u. when switching from phenol to *p*-chlorophenol, while in cellobiose the increase was from 1.3 × 10^−1^ to 1.9 × 10^−1^ a.u. In relative terms, this corresponds to an increase of approximately 65% in chitosan and 52% in cellobiose. Although both increases confirm a more intense interaction for the chlorinated derivative, chitosan again shows a more pronounced response. Even more illustrative is the behavior of the quotient |V|/G: in chitosan, this parameter went from 3.6 in the phenol system to 7.5 in the *p*-chlorophenol system, while in cellobiose, the increase was minimal, from 3.0 to 3.1. This indicates that the introduction of Cl not only increases the local electron density in chitosan but also markedly modifies the energetic nature of intermolecular contacts.

NCI/RDG results lead to supplemental reading. In all four systems, attractive interactions are observed in the region of negative sign(λ2)p, consistent with hydrogen bonds and stabilizing non-covalent contacts. However, the signature for chitosan–*p*-chlorophenol is the most intense and widespread, in accordance with its highest PC, its highest ∇^2^PC, and the maximum value of |V|/G. In contrast, although cellobiose–*p*-chlorophenol also shows an intensification relative to cellobiose–phenol, the change is quantitatively smaller. It suggests a more localized, less cooperative interaction and greater sensitivity to halogenation than that observed in chitosan.

## 3. Materials and Methods

### 3.1. Construction of Molecular Models

To describe, at the molecular level, the adsorption of phenolic pollutants on representative biopolymers, four isolated species were constructed: a fully deacetylated chitosan dimer, a cellobose molecule, phenol, and *p*-chlorophenol. Chitosan dimer was selected as the minimum model of the adsorbent because it simultaneously preserves amino and hydroxyl groups [8]. At the same time, cellobiose was used as a representative cellulose fragment because it preserves the β-(1 → 4) connectivity and the local arrangement of hydroxyl groups responsible for its intermolecular behavior [18,19]. This reduced approximation enables the capture of the immediate chemical adsorption environment without introducing unnecessary structural complexity [17].

### 3.2. Conformational Sampling of Adsorbents

Before the adsorption study, a conformational search was carried out for the isolated chitosan dimer and cellobiose models to identify the most stable starting geometries for complex construction. For the chitosan dimer, several initial conformers were generated by varying the relative orientation of the hydroxyl and amino groups together with the main dihedral arrangement around the β-(1 → 4)-glycosidic linkage. For cellobiose, several initial conformers were generated by modifying the orientations of hydroxyl groups and the principal torsional arrangement around the glycosidic bond, taking into account its intrinsic flexibility [18] and its ability to form intramolecular hydrogen bonds [19]. The initial structures were manually built and subsequently optimized at the selected level of theory. The resulting minima were ranked according to their relative Gibbs free energies, and the lowest-energy conformer of each adsorbent was selected as the reference structure for the adsorption calculations.

Using the optimized geometries of the selected adsorbents and the isolated contaminants, three initial adsorbent–adsorbate arrangements were then constructed for each system. For the chitosan dimer–contaminant pairs, orientation 1 placed the hydroxyl group of phenol or *p*-chlorophenol toward a hydroxyl group of the dimer, orientation 2 directed the contaminant hydroxyl group toward the amino group of chitosan, and orientation 3 corresponded to a mixed arrangement in which the adsorbate was positioned between two functional regions of the dimer to explore possible cooperative stabilization. For the cellobiose–contaminant pairs, orientation 1 placed the hydroxyl group of the contaminant toward a donor/acceptor hydroxyl group of cellobiose, orientation 2 located the adsorbate between two hydroxyl groups of the fragment to probe cooperative recognition, and orientation 3 corresponded to a semi-parallel arrangement of the aromatic ring near the polar surface of cellobiose. In total, twelve initial adsorbent–pollutant complexes were generated, corresponding to four adsorbent–contaminant pairs and three orientations per pair. This protocol enabled direct comparison of hydroxyl–hydroxyl recognition, hydroxyl–amino recognition, and less directional adsorption motifs that involve combined polar and dispersive contributions.

### 3.3. Geometric Optimization of Isolated Species

**Figure 7:**
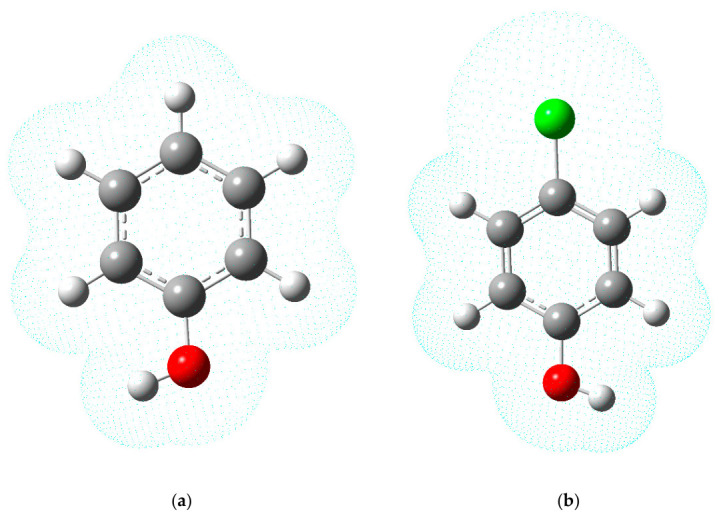
Optimized structures of (**a**) phenol and (**b**) *p*-chlorophenol, showing the spatial distribution of their electronic surfaces in the aqueous phase using the SMD continuous solvation model.

The geometric optimizations and vibrational frequency calculations of the isolated species and the adsorbent–pollutant complexes were carried out using density functional theory (DFT) with the Gaussian 16 program [20], at the ωB97X-D/def2-TZVP level of theory. This functional was selected because it provides a balanced description of hydrogen bonding, long-range interactions, and dispersion-corrected noncovalent contacts in organic systems, all of which are relevant to the present adsorption study. The effect of the aqueous medium was incorporated throughout the computational protocol using the SMD continuum solvation model [21] (Figure 7), so that all geometry optimizations and harmonic frequency calculations were performed directly in solution, rather than using gas-phase-optimized structures followed by single-point solvation corrections. This strategy was adopted to account explicitly for the influence of the polar environment on the relative stability of the isolated species and the adsorbent–adsorbate complexes. Subsequently, vibrational frequency calculations were performed at the same level of theory and under the same solvation conditions to confirm that all optimized structures corresponded to true minima on the potential energy surface [20], as verified by the absence of imaginary frequencies.

### 3.4. Construction of Adsorbent–Pollutant Complexes

From the optimized geometries of the adsorbents and contaminants, initial complexes were constructed for each adsorbent–adsorbate pair. Initial arrangements were manually constructed in GaussView by placing the phenolic hydroxyl group toward the targeted functional region of the adsorbent and varying the relative position of the aromatic ring (Tables S2–S17). For the chitosan–contaminant dimer system, three initial orientations were considered: an arrangement in which the hydroxyl group of the contaminant was oriented towards a hydroxyl group of the dimer, an orientation in which the hydroxyl group of the contaminant pointed towards the amino group of the chitosan, and a mixed arrangement in which the adsorbate was located between two functional groups of the adsorbent to explore a possible cooperative stabilization. For the cellobiose–contaminant system, three orientations were evaluated: a geometry with the hydroxyl group of the contaminant directed towards a donor/acceptor hydroxyl group of the cellobiose, an orientation located between two hydroxyl groups of the fragment to investigate cooperative interaction, and a semi-parallel arrangement of the aromatic ring close to the polar surface of the adsorbent. This strategy is consistent with the need to explore different modes of non-covalent recognition in molecular adsorption studies [16,17]. In total, twelve initial complexes were generated, corresponding to four adsorbent–pollutant pairs and three orientations per pair.

### 3.5. Optimization and Verification of Complexes

All the complexes built were fully optimized at the same level of theory used for the isolated species, to guarantee methodological consistency in the comparison of energies and structures [17]. Subsequently, frequency calculations were performed to confirm that the optimized complexes corresponded to local minima [20] and to obtain the thermal corrections required for the thermodynamic evaluation of the adsorption process.

### 3.6. Calculation of Energy Adsorption Parameters

The relative stability of each complex was evaluated by calculating the adsorption energy (ΔEads), the adsorption enthalpy (ΔHads), and the adsorption free energy (ΔGads), using the following expressions [17]:

ΔEads = Ecomplex − (Eadsorbent + Eadsorbate)(1)

ΔHads = Hcomplex − (Hadsorbent + Hadsorbate)(2)

ΔGads = Gcomplex − (Gadsorbent + Gadsorbate)(3)

These parameters allowed comparison of the relative affinities of phenol and *p*-chlorophenol towards chitosan dimer and cellobiose, as well as the identification of the most favorable geometries in aqueous medium.

### 3.7. Electronic Analysis of Isolated Species and Complexes

To interpret the adsorption mechanism from an electronic perspective, the boundary orbitals, molecular electrostatic potential maps, and charge redistribution for the isolated species and the most stable complexes in each series were analyzed. The analysis of HOMO and LUMO was used to examine qualitative changes in the electron distribution upon complex formation. In contrast, electrostatic potential maps were used to identify the most favorable regions for intermolecular recognition between the adsorbent and the adsorbate.

### 3.8. Analysis of Non-Covalent Interactions

The post-calculation analyses were performed with the Multiwfn program [22], using the wave function files generated from the optimized structures in Gaussian 16 [20]. The most stable geometries obtained for each system were subjected to an NCI/RDG analysis to visualize and characterize the non-covalent interactions responsible for adsorption [23,24]. This treatment enabled differentiation of regions dominated by hydrogen bonds, dispersive contacts, and steric repulsion. In addition, QTAIM analyses were performed on the most representative complexes [25], which allowed us to identify critical binding points between the adsorbent and the contaminant and support the mechanistic interpretation of the observed interactions [26].

### 3.9. Comparative Interpretation Strategy

The interpretation of the results was carried out from two main comparative axes. The first was to evaluate the effect of chlorine substitution at the para position on phenol adsorption, analyzing how the stability, geometry, and nature of the interaction change when switching from phenol to *p*-chlorophenol [16]. The second focused on comparing the performance of chitosan dimer and cellobiose as molecular models of adsorbents to determine the effect of an environment with amino and hydroxyl groups versus one dominated exclusively by hydroxyl groups [8,19].

## 4. Conclusions

This study shows that chitosan and cellobiose share the same primary adsorption motif toward phenol and *p*-chlorophenol: an initial O–H···O interaction between the contaminant hydroxyl group and an adsorbent hydroxyl group. However, the two biopolymers differ significantly in the way they modulate conformational selectivity, local electronic response, and noncovalent interaction strength.

For phenol, both adsorbents showed very similar electron density values at the bond critical point, indicating that a properly oriented hydroxyl network is sufficient to stabilize simple phenolic contaminants with comparable intensity. In contrast, the introduction of chlorine produced a response strongly dependent on the adsorbent microenvironment. In chitosan, the increase in PC, ∇^2^PC, and |V|/G was markedly greater than in cellobiose, and alternative orientations became strongly disfavored. In cellobiose, the same substitution led to a more moderate increase in topological interaction strength, while alternative adsorption geometries remained relatively accessible.

Overall, the results establish the following qualitative order of noncovalent interaction strength:

Chitosan–*p*-chlorophenol > cellobiose–*p*-chlorophenol > chitosan–phenol ≈ cellobiose–phenol

These findings indicate that the main comparative advantage of chitosan does not lie only in the presence of an amino group, but in a functionally more heterogeneous and electronically more adaptable microenvironment, capable of amplifying the noncovalent reorganization induced by aromatic substitution. Accordingly, the adsorption of substituted phenols on biopolymers should be understood as a cooperative process in which a primary polar anchor is complemented by secondary dispersive and electrostatic contributions, whose relative importance depends on both the adsorbent and the adsorbate substituent.

## Figures and Tables

**Table 1:** Energy and orbital boundary parameters of model adsorbents and isolated pollutants.

Scheme	HOMO (eV)	LUMO (eV)	Gap (eV)
Chitosan	−8.9	3.1	12.0
Cellobiose	−9.9	3.0	12.4
Phenol	−8.3	1.5	9.8
*p*-Chlorophenol	−8.2	1.3	9.5

**Table 2:** Relative Gibbs free energies (ΔGrel) and shorter interfragment polar contacts for optimized adsorbent–contaminant complexes. ΔGrel was calculated with respect to the minimum of each adsorbent–pollutant family.

Complex	Description of the Orientation	ΔG (kcal mol^−1^)	Polar Contact 1 (Å)	Polar Contact 2 (Å)
Chitosan–phenol–1	–OH of phenol to –OH of dimer	0.0	2.6	2.4
Chitosan–phenol–2	–OH from phenol to –NH_2_	2.7	3.2	2.5
Chitosan–phenol–3	Mixed orientation	21.2	2.4	1.9
Chitosan–*p*-chlorophenol–1	–OH of *p*-chlorophenol to –OH of dimer	0.0	2.5	2.5
Chitosan–*p*-chlorophenol–2	–OH from *p*-chlorophenol to –NH_2_	43.6	2.4	2.1
Chitosan–*p*-chlorophenol–3	Mixed orientation	46.4	2.2	1.8
Cellobiose–phenol–1	–Phenol OH towards a –OH donor/acceptor of cellobiose	0.0	2.6	2.8
Cellobiose–phenol–2	Orientation between two hydroxyl groups	6.6	2.8	2.2
Cellobiose–phenol–3	Semi-parallel orientation	48.9	2.9	2.8
Cellobiose–*p*-chlorophenol–1	*p*-chlorophenol –OH towards a –OH cellobiose donor/acceptor	0.0	2.2	2.3
Cellobiose–*p*-chlorophenol–2	Orientation between two hydroxyl groups	5.1	3.0	1.9
Cellobiose–*p*-chlorophenol–3	Semi-parallel orientation	3.5	3.2	2.7

**Table 3:** Key interatomic distances for the optimized adsorbent–contaminant complexes are shown in Figure 2 and Figure 3.

Complex	Figure Panel	Orientation Description	ΔGrel (kcal mol^−1^)	Main Interaction Motif	d_1_ (Å)	d_2_ (Å)
Chitosan–phenol–1	Figure 2a	Phenol –OH directed toward a chitosan –OH group	0.0	O–H···O/polar contact	2.6	2.4
Chitosan–phenol–2	Figure 2b	Phenol –OH directed toward the chitosan –NH_2_ group	2.7	O–H···N/polar contact	3.2	2.5
Chitosan–phenol–3	Figure 2c	Mixed orientation between two functional regions	21.2	Mixed polar contacts	2.4	1.9
Chitosan–*p*-Chlorophenol–1	Figure 2d	p-Chlorophenol –OH directed toward a chitosan –OH group	0.0	O–H···O/polar contact	2.5	2.5
Chitosan–*p*-chlorophenol–2	Figure 2e	p-Chlorophenol –OH directed toward the chitosan –NH_2_ group	43.6	O–H···N/polar contact	2.4	2.1
Chitosan–*p*-chlorophenol–3	Figure 2f	Mixed orientation between two functional regions	46.4	Mixed polar contacts	2.2	1.8
Cellobiose–phenol–1	Figure 3a	Phenol –OH directed toward a donor/acceptor –OH group of cellobiose	0.0	O–H···O/polar contact	2.6	2.8
Cellobiose–phenol–2	Figure 3b	Phenol is located between two hydroxyl groups	6.6	O–H···O/cooperative polar contacts	2.8	2.2
Cellobiose–phenol–3	Figure 3c	Semi-parallel orientation near the polar surface	48.9	Weak polar contacts	2.9	2.8
Cellobiose–*p*-chlorophenol–1	Figure 3d	*p*-Chlorophenol –OH directed toward a donor/acceptor –OH group of cellobiose	0.0	O–H···O/polar contact	2.2	2.3
Cellobiose–*p*-chlorophenol–2	Figure 3e	*p*-Chlorophenol is located between two hydroxyl groups	5.1	O–H···O/cooperative polar contacts	3.0	1.9
Cellobiose–*p*-chlorophenol–3	Figure 3f	Semi-parallel orientation near the polar surface	3.5	Weak polar contacts	3.2	2.7

**Table 4:** QTAIM topological parameters at the critical binding point of representative complexes.

Molecular Structure	*PC*	∇^2^PC	H	V	G	|V|/G
Chitosan-phenol(–Phenol OH to dimer –OH)	3.2 × 10^−2^	1.4 × 10^−1^	7.7 × 10^−3^	−2.1 × 10^−2^	2.9 × 10^−2^	3.6 × 10^0^
Chitosan-*p*-chlorophenol(–OH of *p*-chlorophenol to –OH of dimer)	6.3 × 10^−2^	2.4 × 10^−1^	1.2 × 10^−2^	−3.6 × 10^−2^	4.8 × 10^−2^	7.5 × 10^0^
Cellobiose-phenol(–Phenol OH to –OH cellobiose donor/acceptor)	3.2 × 10^−2^	1.3 × 10^−1^	6.1 × 10^−3^	−1.9 × 10^−2^	2.6 × 10^−2^	3.0 × 10^0^
Cellobiose-*p*-chlorophenol(–*p*-chlorophenol OH to –OH cellobiose donor/acceptor)	4.2 × 10^−2^	1.9 × 10^−1^	8.7 × 10^−3^	−3.1 × 10^−2^	3.9 × 10^−2^	3.1 × 10^0^

## Data Availability

The original contributions presented in this study are included in the article. Further inquiries can be directed to the corresponding authors.

## Supplementary Materials

The following supporting information can be downloaded at: https://www.mdpi.com/article/10.3390/molecules31111871/s1.
