# Supplementary material for: Comparative Adsorption of Phenol and p-Chlorophenol on a Chitosan–Cellobiose Dimer in an Aqueous Medium: A DFT Study of Hydrogen Bonding and Noncovalent Interactions
Source: Molecules. 2026 May 29;31(11):1871. doi: 10.3390/molecules31111871 (PMC13258039; doi:10.3390/molecules31111871)
Supplement: Supplementary file 1 [file molecules-31-01871-s001.zip › molecules-4310652-supplementary.pdf]

Table S1. Energy and orbital boundary parameters of model adsorbents and isolated pollutants.

Scheme	E.system (Hartree)	Enthalpy (Hartree)	Gibbs (Hartree) Free Energy
Chitosan	-1257.97764	-1257.976696	-1258.056657
Cellobiose	-1297.732276	-1297.731332	-1297.811667
Phenol	-307.378759	-307.377814	-307.413171
p-Chlorophenol	-766.998165	-766.997221	-767.035926

Table S2. Cellobiose coordinates

C	-5.01831	-0.38878	-0.33795
C	-3.50321	-0.38878	-0.33795
C	-2.95128	1.0223	-0.33795
C	-5.01607	1.8275	0.82222
C	-5.56887	0.41688	0.82093
H	-3.13066	-0.93441	0.56861
H	-5.391	0.04425	-1.30334
H	-5.38792	2.3741	-0.0841
H	-5.30286	-0.08884	1.78624
H	-3.19455	1.54256	-1.33045
O	-1.91102	1.49357	-1.23944
C	-0.30363	1.57939	-1.40594
C	0.53029	2.77747	-0.9998
C	0.52804	0.56209	-2.16091
H	-0.12384	3.53775	-0.49907
C	1.21044	1.19041	-3.35888
H	-0.12758	-0.28087	-2.50153
C	2.04354	2.38932	-2.95388
H	1.86492	0.43032	-3.85935
H	2.48194	2.86798	-3.86782
O	3.13069	1.95697	-2.13164
H	3.96219	2.1791	-2.55692
O	-6.99576	0.4616	0.73809
H	-7.37509	0.05259	1.51938
O	0.22201	1.59804	-4.30848
H	0.4963	1.3342	-5.18982
O	1.51579	0.01189	-1.28539
H	1.70903	-0.89119	-1.54752
H	-0.6999	1.11834	-0.52543
O	1.21334	3.40595	-2.1973
C	1.59408	2.33697	0.0229
H	1.11286	2.02594	0.92655
H	2.15927	1.52211	-0.37892
O	2.46964	3.43263	0.30186
H	2.78361	3.36955	1.20687

O	-3.50094	1.82684	0.82271
O	-3.02446	-1.0902	-1.48847
H	-3.36065	-1.98936	-1.47848
O	-5.49747	-1.73367	-0.25694
H	-6.29607	-1.82154	-0.78242
C	-5.53169	2.58232	2.06157
H	-6.56921	2.81074	1.93399
H	-5.40541	1.97024	2.93008
O	-4.79265	3.79596	2.22218
H	-5.25701	4.37584	2.83022

Table S3. Chitosan coordinates

C	-5.01831448	-0.38877641	-0.33795060
C	-3.50320848	-0.38877641	-0.33795060
C	-2.95127748	1.02230159	-0.33795060
C	-5.01606548	1.82749959	0.82222140
C	-5.56886548	0.41687859	0.82092540
H	-3.13065948	-0.93441041	0.56861140
H	-5.39100248	0.04425259	-1.30333560
H	-5.38792048	2.37410059	-0.08410160
H	-5.30286148	-0.08884341	1.78624040
H	-3.21902187	1.50321034	-1.25552333
C	-0.30362838	1.57938780	-1.40593866
C	0.53028776	2.77747193	-0.99980170
C	0.52803863	0.56209039	-2.16090828
C	2.04353590	2.38931914	-2.95388202
O	-6.99576323	0.46160484	0.73808596
H	-7.37509346	0.05259209	1.51937793
O	-3.50094048	1.82683859	0.82271040
O	-5.49747323	-1.73367198	-0.25693947
H	-6.29606678	-1.82153504	-0.78241870
C	-5.53168963	2.58231773	2.06156853
H	-6.56920914	2.81073801	1.93398546
H	-5.40541246	1.97023752	2.93007983
O	-4.79265470	3.79596183	2.22217787
H	-5.25701058	4.37584222	2.83021763
H	-1.10762907	1.90781209	-2.03093495
O	-1.52594556	0.96754431	-0.23631129
N	-3.01107278	-1.10982509	-1.52065566
H	-2.74669031	-2.03807780	-1.25902840
H	-3.73378766	-1.15102134	-2.21057310
H	1.27406675	2.46413299	-0.29729795
O	-0.31729311	3.74924467	-0.38160723
H	0.21757353	4.46814705	-0.03708286
C	1.21334309	3.40595067	-2.19729745
H	0.53826444	3.79031145	-2.93312053
H	1.27187977	0.16126598	-1.50444796

O	1.21044191	1.19041489	-3.35888096
O	2.60279780	2.99993275	-4.11976244
H	3.49669084	2.67639371	-4.25347004
H	2.84096922	2.05722999	-2.32244169
C	-0.38686622	-0.57901518	-2.64301532
H	-0.62860005	-1.21533935	-1.81745109
H	-1.28612527	-0.16698488	-3.05101555
N	1.99469228	4.51136516	-1.62418991
H	2.37102279	4.43170713	-0.70113523
O	0.28845802	-1.33885556	-3.64873883
H	0.00761951	-2.25558282	-3.60035647
H	2.14989183	5.34300583	-2.15737552

Table S4. Phenol coordinates

C	0.94260800	0.75548900	0.64821900
C	2.33088000	0.80127400	0.66190900
C	2.97093700	2.02912300	0.56787200
C	2.23805300	3.20219000	0.46192900
C	0.84931000	3.14388700	0.45173000
C	0.19792500	1.91954700	0.54335700
H	0.43033700	-0.19599700	0.72125200
H	2.90715900	-0.11099100	0.74560200
H	4.05279500	2.08021000	0.57716900
H	2.73572000	4.16256000	0.38914200
H	-0.88460300	1.89093200	0.53358800
O	0.07696100	4.26659000	0.35504700
H	0.64113600	5.04534700	0.31833400

Table S5. P-Clorophenol coordinates

C	0.93445456	0.75336351	0.63375075
C	2.31830813	0.81193915	0.64293528
C	2.98175386	2.02240773	0.53192014
C	2.25287098	3.20214598	0.47491203
C	0.86350300	3.15045275	0.48667325
C	0.21032807	1.92607428	0.51751713
H	0.42061296	-0.19397143	0.73293136
H	4.06320819	2.04979169	0.50206428
H	2.77062516	4.15381467	0.43808749
H	-0.87032280	1.91038173	0.46585155
O	0.07515141	4.26666148	0.52025886
H	0.56530934	5.02985953	0.21442508
Cl	3.23339844	-0.66474319	0.81461173

Table S6. Chitosano-clorophenol-1 coordinates

C	-5.16560800	-0.24770300	-0.32736400
C	-3.73922200	-0.57373700	-0.76561400
C	-2.91729700	0.71080800	-0.78367100
C	-4.31070500	1.80277600	0.79162600

C	-5.21738900	0.58022200	0.94580800
H	-3.28997300	-1.23665800	-0.02135900
H	-5.63842300	0.34489800	-1.12396300
H	-4.69829700	2.43475700	-0.01961000
H	-4.85624400	-0.02685900	1.78308400
H	-3.28687100	1.38012900	-1.57166600
C	-0.82374300	1.24987100	-1.85104200
C	-0.58699600	2.64492600	-1.28428100
C	0.51274100	0.52339500	-2.04891000
C	1.75198700	2.52941000	-2.14608100
O	-6.57385100	0.94224800	1.14892300
H	-6.71902800	1.10069700	2.08468000
O	-3.00359700	1.35902500	0.46884900
O	-5.86323500	-1.47116900	-0.16857100
H	-6.76849200	-1.25922100	0.07819000
C	-4.21846900	2.61919300	2.05593900
H	-5.22912300	2.85433400	2.40193700
H	-3.71626600	2.02682700	2.82860800
O	-3.49613300	3.80964300	1.78414800
H	-3.41498400	4.30202400	2.60425800
H	-1.34030600	1.33731200	-2.81402500
O	-1.57416700	0.41427100	-0.98029900
N	-3.67631300	-1.22531800	-2.06576300
H	-4.18347700	-2.10026500	-2.01802300
H	-4.16684400	-0.65093100	-2.74418300
H	-0.27655600	2.52743900	-0.23632300
O	-1.78295000	3.40198200	-1.35071600
H	-1.51780100	4.31619700	-1.18736100
C	0.51161400	3.39769100	-2.03120300
H	0.16324900	3.58705200	-3.05041500
H	0.94328300	0.32204100	-1.05844800
O	1.40996100	1.32162600	-2.79903200
O	2.69830800	3.18506600	-2.93137400
H	3.55740000	2.77895100	-2.78444200
H	2.16225600	2.29774400	-1.15274200
C	0.33925700	-0.78113300	-2.78879700
H	-0.43848500	-1.36910500	-2.29830700
H	0.02092800	-0.56821100	-3.81523300
N	0.71133000	4.67769200	-1.37093900
H	1.16863200	4.52902800	-0.47721000
O	1.57603000	-1.47760600	-2.77886700
H	1.46023600	-2.29425000	-3.27043900
H	1.32505700	5.25927700	-1.92647000
C	0.02847495	8.02714438	4.23179606
C	1.18851279	7.96607284	3.47705143
C	1.28054149	7.12727881	2.38015070
C	0.19540487	6.33955484	2.03072436
C	-0.97366845	6.39753059	2.77957415

C	-1.05492108	7.24002572	3.88160315
H	-0.03513749	8.68525273	5.08799980
H	2.19017389	7.08414723	1.79634589
H	0.25577837	5.68052855	1.17285562
H	-1.96885050	7.27805298	4.46019156
O	-2.07053905	5.64845791	2.47334769
H	-1.89975909	5.12853919	1.68135334
Cl	2.55366419	8.96325824	3.91487867

Table S7. Chitosano-clorophenol-2 coordinates

C	-5.16560800	-0.24770300	-0.32736400
C	-3.73922200	-0.57373700	-0.76561400
C	-2.91729700	0.71080800	-0.78367100
C	-4.31070500	1.80277600	0.79162600
C	-5.21738900	0.58022200	0.94580800
H	-3.28997300	-1.23665800	-0.02135900
H	-5.63842300	0.34489800	-1.12396300
H	-4.69829700	2.43475700	-0.01961000
H	-4.85624400	-0.02685900	1.78308400
H	-3.28687100	1.38012900	-1.57166600
C	-0.82374300	1.24987100	-1.85104200
C	-0.58699100	2.64492400	-1.28427700
C	0.51274100	0.52339500	-2.04891000
C	1.75199100	2.52941600	-2.14609000
O	-6.57385100	0.94224800	1.14892300
H	-6.71902800	1.10069700	2.08468000
O	-3.00359700	1.35902500	0.46884900
O	-5.86323500	-1.47116900	-0.16857100
H	-6.76849200	-1.25922100	0.07819000
C	-4.21846900	2.61919300	2.05593900
H	-5.22912300	2.85433400	2.40193700
H	-3.71626600	2.02682700	2.82860800
O	-3.49613300	3.80964300	1.78414800
H	-3.41498400	4.30202400	2.60425800
H	-1.34030600	1.33731200	-2.81402500
O	-1.57416700	0.41427100	-0.98029900
N	-3.67631300	-1.22531800	-2.06576300
H	-4.18347700	-2.10026500	-2.01802300
H	-4.16684400	-0.65093100	-2.74418300
H	-0.27655600	2.52743900	-0.23632300
O	-1.78295000	3.40198200	-1.35071600
H	-1.50227200	4.31623900	-1.20364500
C	0.51162100	3.39769300	-2.03120800
H	0.16324600	3.58704800	-3.05041500
H	0.94328300	0.32204100	-1.05844800
O	1.40996100	1.32162600	-2.79903200
O	2.69830800	3.18506600	-2.93137400
H	3.55740000	2.77895100	-2.78444200

H	2.16225600	2.29774400	-1.15274200
C	0.33925700	-0.78113300	-2.78879700
H	-0.43848500	-1.36910500	-2.29830700
H	0.02092800	-0.56821100	-3.81523300
N	0.71134500	4.67775200	-1.37092300
H	1.21672600	4.54576800	-0.50732700
O	1.57603000	-1.47760600	-2.77886700
H	1.46023600	-2.29425000	-3.27043900
H	1.23043400	5.31281700	-1.95412200
C	2.53331200	9.48316400	0.27324000
C	1.82820600	10.09776900	1.29507200
C	0.67580000	9.52793400	1.80740500
C	0.22284800	8.32393000	1.29229400
C	0.92622900	7.69824200	0.27012500
C	2.08006100	8.28033400	-0.24027800
H	3.43306700	9.93544400	-0.12188400
H	0.12989700	10.01444400	2.60453600
H	-0.67721600	7.86791900	1.68705600
H	2.62106700	7.78691600	-1.03735300
O	0.52611500	6.51034400	-0.26559300
H	-0.30265900	6.21384900	0.12210200
Cl	2.40281900	11.61236700	1.94739400

Table S8. Chitosano-clorophenol-3 coordinates

C	-5.16560800	-0.24770300	-0.32736400
C	-3.73922200	-0.57373700	-0.76561400
C	-2.91729700	0.71080800	-0.78367100
C	-4.31070500	1.80277600	0.79162600
C	-5.21738900	0.58022200	0.94580800
H	-3.28997300	-1.23665800	-0.02135900
H	-5.63842300	0.34489800	-1.12396300
H	-4.69829700	2.43475700	-0.01961000
H	-4.85624400	-0.02685900	1.78308400
H	-3.28687100	1.38012900	-1.57166600
C	-0.82374300	1.24987100	-1.85104200
C	-0.58699600	2.64492600	-1.28428100
C	0.51274100	0.52339500	-2.04891000
C	1.75198700	2.52941000	-2.14608100
O	-6.57385100	0.94224800	1.14892300
H	-6.71902800	1.10069700	2.08468000
O	-3.00359700	1.35902500	0.46884900
O	-5.86323500	-1.47116900	-0.16857100
H	-6.76849200	-1.25922100	0.07819000
C	-4.21846900	2.61919300	2.05593900
H	-5.22912300	2.85433400	2.40193700
H	-3.71626600	2.02682700	2.82860800
O	-3.49613300	3.80964300	1.78414800

H	-3.41498400	4.30202400	2.60425800
H	-1.34030600	1.33731200	-2.81402500
O	-1.57416700	0.41427100	-0.98029900
N	-3.67631300	-1.22531800	-2.06576300
H	-4.18347700	-2.10026500	-2.01802300
H	-4.16684400	-0.65093100	-2.74418300
H	-0.27655600	2.52743900	-0.23632300
O	-1.78295000	3.40198200	-1.35071600
H	-1.51780100	4.31619700	-1.18736100
C	0.51161400	3.39769100	-2.03120300
H	0.16324900	3.58705200	-3.05041500
H	0.94328300	0.32204100	-1.05844800
O	1.40996100	1.32162600	-2.79903200
O	2.69830800	3.18506600	-2.93137400
H	3.55740000	2.77895100	-2.78444200
H	2.16225600	2.29774400	-1.15274200
C	0.33925700	-0.78113300	-2.78879700
H	-0.43848500	-1.36910500	-2.29830700
H	0.02092800	-0.56821100	-3.81523300
N	0.71133000	4.67769200	-1.37093900
H	1.16863200	4.52902800	-0.47721000
O	1.57603000	-1.47760600	-2.77886700
H	1.46023600	-2.29425000	-3.27043900
H	1.32505700	5.25927700	-1.92647000
C	-6.42300935	-6.37031531	-2.10306468
C	-7.05755470	-6.43540982	-3.33276866
C	-7.26448222	-5.29440388	-4.08809799
C	-6.82794033	-4.07000146	-3.60809883
C	-6.18670316	-3.99593400	-2.37757109
C	-5.98712343	-5.14672548	-1.62476644
H	-6.26547457	-7.26693821	-1.51869481
H	-7.76185645	-5.35300662	-5.04690146
H	-6.98310947	-3.17052734	-4.19185240
H	-5.48794299	-5.07988415	-0.66673265
O	-5.73273005	-2.81745528	-1.86463254
H	-5.90705326	-2.10273252	-2.48553439
Cl	-7.60245681	-7.97968177	-3.93941154

Table S9. Chitosano-phenol-1 coordinates

C	-5.16560800	-0.24770300	-0.32736400
C	-3.73922200	-0.57373700	-0.76561400
C	-2.91729700	0.71080800	-0.78367100
C	-4.31070500	1.80277600	0.79162600
C	-5.21738900	0.58022200	0.94580800
H	-3.28997300	-1.23665800	-0.02135900
H	-5.63842300	0.34489800	-1.12396300
H	-4.69829700	2.43475700	-0.01961000

H	-4.85624400	-0.02685900	1.78308400
H	-3.28687100	1.38012900	-1.57166600
C	-0.82374300	1.24987100	-1.85104200
C	-0.58699600	2.64492600	-1.28428100
C	0.51274100	0.52339500	-2.04891000
C	1.75198700	2.52941000	-2.14608100
O	-6.57385100	0.94224800	1.14892300
H	-6.71902800	1.10069700	2.08468000
O	-3.00359700	1.35902500	0.46884900
O	-5.86323500	-1.47116900	-0.16857100
H	-6.76849200	-1.25922100	0.07819000
C	-4.21846900	2.61919300	2.05593900
H	-5.22912300	2.85433400	2.40193700
H	-3.71626600	2.02682700	2.82860800
O	-3.49613300	3.80964300	1.78414800
H	-3.41498400	4.30202400	2.60425800
H	-1.34030600	1.33731200	-2.81402500
O	-1.57416700	0.41427100	-0.98029900
N	-3.67631300	-1.22531800	-2.06576300
H	-4.18347700	-2.10026500	-2.01802300
H	-4.16684400	-0.65093100	-2.74418300
H	-0.27655600	2.52743900	-0.23632300
O	-1.78295000	3.40198200	-1.35071600
H	-1.51780100	4.31619700	-1.18736100
C	0.51161400	3.39769100	-2.03120300
H	0.16324900	3.58705200	-3.05041500
H	0.94328300	0.32204100	-1.05844800
O	1.40996100	1.32162600	-2.79903200
O	2.69830800	3.18506600	-2.93137400
H	3.55740000	2.77895100	-2.78444200
H	2.16225600	2.29774400	-1.15274200
C	0.33925700	-0.78113300	-2.78879700
H	-0.43848500	-1.36910500	-2.29830700
H	0.02092800	-0.56821100	-3.81523300
N	0.71133000	4.67769200	-1.37093900
H	1.16863200	4.52902800	-0.47721000
O	1.57603000	-1.47760600	-2.77886700
H	1.46023600	-2.29425000	-3.27043900
H	1.32505700	5.25927700	-1.92647000
C	0.02847495	8.02714438	4.23179606
C	1.18851279	7.96607284	3.47705143
C	1.28054149	7.12727881	2.38015070
C	0.19540487	6.33955484	2.03072436
C	-0.97366845	6.39753059	2.77957415
C	-1.05492108	7.24002572	3.88160315
H	-0.03513749	8.68525273	5.08799980
H	2.19017389	7.08414723	1.79634589
H	0.25577837	5.68052855	1.17285562

H	-1.96885050	7.27805298	4.46019156
O	-2.07053905	5.64845791	2.47334769
H	-1.89975909	5.12853919	1.68135334
H	2.02495385	8.57705768	3.74531228

Table S10. Chitosano-phenol-2 coordinates

C	-5.16560800	-0.24770300	-0.32736400
C	-3.73922200	-0.57373700	-0.76561400
C	-2.91729700	0.71080800	-0.78367100
C	-4.31070800	1.80277900	0.79162100
C	-5.21738900	0.58022200	0.94580800
H	-3.28997300	-1.23665800	-0.02135900
H	-5.63842300	0.34489800	-1.12396300
H	-4.69829700	2.43475700	-0.01961000
H	-4.85624400	-0.02685900	1.78308400
H	-3.28687100	1.38012900	-1.57166600
C	-0.82363900	1.24981400	-1.85094600
C	-0.58755500	2.64525100	-1.28477700
C	0.51274100	0.52339500	-2.04891000
C	1.75222900	2.52982600	-2.14661300
O	-6.57385100	0.94224800	1.14892300
H	-6.71902800	1.10069700	2.08468000
O	-3.00359700	1.35902500	0.46884900
O	-5.86323500	-1.47116900	-0.16857100
H	-6.76849200	-1.25922100	0.07819000
C	-4.21846900	2.61919300	2.05593900
H	-5.22912300	2.85433400	2.40193700
H	-3.71626600	2.02682700	2.82860800
O	-3.49613300	3.80964300	1.78414800
H	-3.41498400	4.30202400	2.60425800
H	-1.34030600	1.33731200	-2.81402500
O	-1.57416700	0.41427100	-0.98029900
N	-3.67631300	-1.22531800	-2.06576300
H	-4.18347700	-2.10026500	-2.01802300
H	-4.16684400	-0.65093100	-2.74418300
H	-0.27664500	2.52761500	-0.23627700
O	-1.78297700	3.40193100	-1.35080800
H	-1.51780100	4.31619700	-1.18736100
C	0.51275800	3.39706900	-2.03060000
H	0.16251500	3.58699100	-3.05017600
H	0.94328300	0.32204100	-1.05844800
O	1.40994100	1.32158500	-2.79894600
O	2.69833000	3.18509100	-2.93132700
H	3.55740000	2.77895100	-2.78444200
H	2.16217800	2.29763200	-1.15273600
C	0.33925700	-0.78113300	-2.78879700
H	-0.43848500	-1.36910500	-2.29830700

H	0.02092800	-0.56821100	-3.81523300
N	0.70820300	4.67542800	-1.37015400
H	1.20354200	4.54565700	-0.49205800
O	1.57603000	-1.47760600	-2.77886700
H	1.46023600	-2.29425000	-3.27043900
H	1.31925800	5.26514700	-1.92163100
C	6.34226400	6.64144800	-1.41841600
C	6.92248100	7.34065600	-0.36419400
C	6.13903900	7.69944600	0.72702300
C	4.78892400	7.36894500	0.77104600
C	4.21849500	6.67217800	-0.29176600
C	4.99481700	6.30553300	-1.38849200
H	6.94257200	6.35492000	-2.27561200
H	6.57893500	8.24285200	1.55689700
H	4.17767500	7.64911700	1.62388000
H	4.53363300	5.76115500	-2.20635000
O	2.89826100	6.32179500	-0.31486300
H	2.45647300	6.62528100	0.48689800
H	7.97490200	7.60210500	-0.39284800

Table S11. Chitosano-phenol-3 coordinates

C	-5.16560800	-0.24770300	-0.32736400
C	-3.73922200	-0.57373700	-0.76561400
C	-2.91729700	0.71080800	-0.78367100
C	-4.31070500	1.80277600	0.79162600
C	-5.21738900	0.58022200	0.94580800
H	-3.28997300	-1.23665800	-0.02135900
H	-5.63842300	0.34489800	-1.12396300
H	-4.69829700	2.43475700	-0.01961000
H	-4.85624400	-0.02685900	1.78308400
H	-3.28687100	1.38012900	-1.57166600
C	-0.82374300	1.24987100	-1.85104200
C	-0.58699600	2.64492600	-1.28428100
C	0.51274100	0.52339500	-2.04891000
C	1.75198700	2.52941000	-2.14608100
O	-6.57385100	0.94224800	1.14892300
H	-6.71902800	1.10069700	2.08468000
O	-3.00359700	1.35902500	0.46884900
O	-5.86323500	-1.47116900	-0.16857100
H	-6.76849200	-1.25922100	0.07819000
C	-4.21846900	2.61919300	2.05593900
H	-5.22912300	2.85433400	2.40193700
H	-3.71626600	2.02682700	2.82860800
O	-3.49613300	3.80964300	1.78414800
H	-3.41498400	4.30202400	2.60425800
H	-1.34030600	1.33731200	-2.81402500
O	-1.57416700	0.41427100	-0.98029900

N	-3.67631300	-1.22531800	-2.06576300
H	-4.18347700	-2.10026500	-2.01802300
H	-4.16684400	-0.65093100	-2.74418300
H	-0.27655600	2.52743900	-0.23632300
O	-1.78295000	3.40198200	-1.35071600
H	-1.51780100	4.31619700	-1.18736100
C	0.51161400	3.39769100	-2.03120300
H	0.16324900	3.58705200	-3.05041500
H	0.94328300	0.32204100	-1.05844800
O	1.40996100	1.32162600	-2.79903200
O	2.69830800	3.18506600	-2.93137400
H	3.55740000	2.77895100	-2.78444200
H	2.16225600	2.29774400	-1.15274200
C	0.33925700	-0.78113300	-2.78879700
H	-0.43848500	-1.36910500	-2.29830700
H	0.02092800	-0.56821100	-3.81523300
N	0.71133000	4.67769200	-1.37093900
H	1.16863200	4.52902800	-0.47721000
O	1.57603000	-1.47760600	-2.77886700
H	1.46023600	-2.29425000	-3.27043900
H	1.32505700	5.25927700	-1.92647000
C	-6.42300935	-6.37031531	-2.10306468
C	-7.05755470	-6.43540982	-3.33276866
C	-7.26448222	-5.29440388	-4.08809799
C	-6.82794033	-4.07000146	-3.60809883
C	-6.18670316	-3.99593400	-2.37757109
C	-5.98712343	-5.14672548	-1.62476644
H	-6.26547457	-7.26693821	-1.51869481
H	-7.76185645	-5.35300662	-5.04690146
H	-6.98310947	-3.17052734	-4.19185240
H	-5.48794299	-5.07988415	-0.66673265
O	-5.73273005	-2.81745528	-1.86463254
H	-5.90705326	-2.10273252	-2.48553439
H	-7.39142133	-7.38159972	-3.70446444

Table S12. Cellobiose -clorophenol-1 coordinates

C	-4.72188900	-0.36625300	-0.57506200
C	-3.37257500	-0.10629500	-1.22279200
C	-3.12499400	1.39840200	-1.31216800
C	-4.53913300	1.85133300	0.54180900
C	-4.85418400	0.36664300	0.74972400
H	-2.59589700	-0.56888500	-0.60269200
H	-5.50553700	0.00502900	-1.24825300
H	-5.29049700	2.28025400	-0.13511400
H	-4.13973400	-0.05421400	1.46530900
H	-3.85163100	1.84005200	-2.00370100
O	-1.87783400	1.73875500	-1.82650600

C	-0.73574900	1.34478300	-1.06477800
C	0.08449200	2.59665700	-0.76540100
C	0.04093300	0.27676200	-1.83907600
H	-0.62620300	3.39845200	-0.56007100
C	0.86709700	0.85895300	-2.98952100
H	-0.68665500	-0.41174900	-2.27625700
C	1.60471200	2.14487700	-2.61072500
H	1.61668300	0.11793600	-3.28444900
H	1.87335900	2.67885400	-3.52687500
O	2.76621000	1.79565600	-1.90049700
H	3.24149900	2.60682500	-1.69487400
O	-6.18212600	0.16882700	1.20234600
H	-6.19807800	0.22533500	2.16070300
O	-0.02624900	1.10886300	-4.05895300
H	0.48302300	1.40614600	-4.81810100
O	0.85763800	-0.40115300	-0.90197200
H	1.34610700	-1.08667600	-1.36628500
H	-1.04525200	0.90830100	-0.11509200
O	0.79477400	3.05997500	-1.91468500
C	0.95506800	2.44756200	0.47214800
H	0.29226000	2.19226200	1.30625000
H	1.68699900	1.64953100	0.35596600
O	1.60158700	3.68866500	0.71085700
H	2.19843700	3.57284100	1.45378000
O	-3.24918400	1.98539900	-0.03749700
O	-3.32856700	-0.62177600	-2.53849400
H	-3.50065300	-1.56677400	-2.49114200
O	-4.84871600	-1.76768600	-0.41588600
H	-5.74327700	-1.95260800	-0.11581300
C	-4.53407000	2.62250100	1.83752100
H	-5.47024500	2.42841600	2.36861600
H	-3.70291300	2.27103500	2.45870800
O	-4.40099100	4.00520600	1.55140500
H	-4.36718000	4.47904800	2.38587900
C	4.39390155	8.33366412	3.75176874
C	3.79036767	9.48664997	3.26601848
C	2.75155400	9.37626160	2.35233929
C	2.31508492	8.13069787	1.92514518
C	2.92520684	6.98403775	2.42015245
C	3.96770186	7.08289962	3.33401065
H	5.20541458	8.40541198	4.46547726
H	2.27157402	10.26656592	1.96492417
H	1.50330583	8.04187005	1.21211975
H	4.43293985	6.18010996	3.70991697
O	2.53342312	5.73194726	2.03922264
H	1.79630804	5.79667707	1.42393804
Cl	4.33687334	11.07064347	3.80443851

Table S13. Cellobiose -clorophenol-2 coordinates

C	-4.72188900	-0.36625300	-0.57506200
C	-3.37257500	-0.10629500	-1.22279200
C	-3.12499400	1.39840200	-1.31216800
C	-4.53913300	1.85133300	0.54180900
C	-4.85418400	0.36664300	0.74972400
H	-2.59589700	-0.56888500	-0.60269200
H	-5.50553700	0.00502900	-1.24825300
H	-5.29049700	2.28025400	-0.13511400
H	-4.13973400	-0.05421400	1.46530900
H	-3.85163100	1.84005200	-2.00370100
O	-1.87783400	1.73875500	-1.82650600
C	-0.73574900	1.34478300	-1.06477800
C	0.08449200	2.59665700	-0.76540100
C	0.04093300	0.27676200	-1.83907600
H	-0.62620300	3.39845200	-0.56007100
C	0.86709700	0.85895300	-2.98952100
H	-0.68665500	-0.41174900	-2.27625700
C	1.60471200	2.14487700	-2.61072500
H	1.61668300	0.11793600	-3.28444900
H	1.87335900	2.67885400	-3.52687500
O	2.76621000	1.79565600	-1.90049700
H	3.24149900	2.60682500	-1.69487400
O	-6.18212600	0.16882700	1.20234600
H	-6.19807800	0.22533500	2.16070300
O	-0.02624900	1.10886300	-4.05895300
H	0.48302300	1.40614600	-4.81810100
O	0.85763800	-0.40115300	-0.90197200
H	1.34610700	-1.08667600	-1.36628500
H	-1.04525200	0.90830100	-0.11509200
O	0.79477400	3.05997500	-1.91468500
C	0.95506800	2.44756200	0.47214800
H	0.29226000	2.19226200	1.30625000
H	1.68699900	1.64953100	0.35596600
O	1.60158700	3.68866500	0.71085700
H	2.19843700	3.57284100	1.45378000
O	-3.24918400	1.98539900	-0.03749700
O	-3.32856700	-0.62177600	-2.53849400
H	-3.50065300	-1.56677400	-2.49114200
O	-4.84871600	-1.76768600	-0.41588600
H	-5.74327700	-1.95260800	-0.11581300
C	-4.53407000	2.62250100	1.83752100
H	-5.47024500	2.42841600	2.36861600
H	-3.70291300	2.27103500	2.45870800
O	-4.40099100	4.00520600	1.55140500
H	-4.36718000	4.47904800	2.38587900
C	6.38046773	6.66164579	0.83267149

C	6.31506779	7.96586069	0.35903750
C	5.27603548	8.33039680	-0.48574317
C	4.30921936	7.40722700	-0.85648994
C	4.38391044	6.10572746	-0.37417201
C	5.42118240	5.72922056	0.47082254
H	7.18577690	6.36386726	1.49288820
H	5.21223256	9.34370158	-0.86291110
H	3.49620487	7.68978364	-1.51561824
H	5.46553380	4.71127617	0.83770576
O	3.45728758	5.15670671	-0.70137574
H	2.78510638	5.54358584	-1.27111314
Cl	7.53659416	9.14366357	0.82621381

Table S14. Cellobiose -clorophenol-3 coordinates

C	-4.72188900	-0.36625300	-0.57506200
C	-3.37257500	-0.10629500	-1.22279200
C	-3.12499400	1.39840200	-1.31216800
C	-4.53913300	1.85133300	0.54180900
C	-4.85418400	0.36664300	0.74972400
H	-2.59589700	-0.56888500	-0.60269200
H	-5.50553700	0.00502900	-1.24825300
H	-5.29049700	2.28025400	-0.13511400
H	-4.13973400	-0.05421400	1.46530900
H	-3.85163100	1.84005200	-2.00370100
O	-1.87783400	1.73875500	-1.82650600
C	-0.73574900	1.34478300	-1.06477800
C	0.08449000	2.59665800	-0.76540000
C	0.04093300	0.27676200	-1.83907600
H	-0.62620300	3.39845200	-0.56007100
C	0.86709700	0.85895300	-2.98952100
H	-0.68665500	-0.41174900	-2.27625700
C	1.60471200	2.14487700	-2.61072500
H	1.61668300	0.11793600	-3.28444900
H	1.87335900	2.67885400	-3.52687500
O	2.76621000	1.79565600	-1.90049700
H	3.23329700	2.60241300	-1.66110000
O	-6.18212600	0.16882700	1.20234600
H	-6.19807800	0.22533500	2.16070300
O	-0.02624900	1.10886300	-4.05895300
H	0.48302300	1.40614600	-4.81810100
O	0.85763800	-0.40115300	-0.90197200
H	1.34610700	-1.08667600	-1.36628500
H	-1.04525200	0.90830100	-0.11509200
O	0.79477400	3.05997500	-1.91468500
C	0.95504700	2.44757500	0.47213600
H	0.29224600	2.19227100	1.30624200
H	1.68699900	1.64953200	0.35596100

O	1.60173100	3.68857900	0.71095400
H	2.03255800	3.64001300	1.56759700
O	-3.24918400	1.98539900	-0.03749700
O	-3.32856700	-0.62177600	-2.53849400
H	-3.50065300	-1.56677400	-2.49114200
O	-4.84871600	-1.76768600	-0.41588600
H	-5.74327700	-1.95260800	-0.11581300
C	-4.53407000	2.62250100	1.83752100
H	-5.47024500	2.42841600	2.36861600
H	-3.70291300	2.27103500	2.45870800
O	-4.40099100	4.00520600	1.55140500
H	-4.36718000	4.47904800	2.38587900
C	3.17728700	1.07623200	2.55716100
C	2.97684100	-0.29766000	2.59988200
C	3.57538300	-1.10157300	1.63988200
C	4.36737200	-0.54551100	0.64591100
C	4.56261200	0.83036500	0.61495000
C	3.96640000	1.64514300	1.57016500
H	2.71641600	1.71519600	3.30040000
H	3.42792000	-2.17439100	1.66059600
H	4.83660700	-1.17146300	-0.10461100
H	4.12780900	2.71536500	1.53196300
O	5.33637600	1.43186300	-0.33679600
H	5.63458300	0.77833800	-0.97710200
Cl	1.97507300	-1.01122900	3.85880100

Table S15. Cellobiose -phenol-1 coordinates

C	-4.72188900	-0.36625300	-0.57506200
C	-3.37257500	-0.10629500	-1.22279200
C	-3.12499400	1.39840200	-1.31216800
C	-4.53913300	1.85133300	0.54180900
C	-4.85418400	0.36664300	0.74972400
H	-2.59589700	-0.56888500	-0.60269200
H	-5.50553700	0.00502900	-1.24825300
H	-5.29049700	2.28025400	-0.13511400
H	-4.13973400	-0.05421400	1.46530900
H	-3.85163100	1.84005200	-2.00370100
O	-1.87783400	1.73875500	-1.82650600
C	-0.73574900	1.34478300	-1.06477800
C	0.08449200	2.59665700	-0.76540100
C	0.04093300	0.27676200	-1.83907600
H	-0.62620300	3.39845200	-0.56007100
C	0.86709700	0.85895300	-2.98952100
H	-0.68665500	-0.41174900	-2.27625700
C	1.60471200	2.14487700	-2.61072500
H	1.61668300	0.11793600	-3.28444900
H	1.87335900	2.67885400	-3.52687500

O	2.76621000	1.79565600	-1.90049700
H	3.24149900	2.60682500	-1.69487400
O	-6.18212600	0.16882700	1.20234600
H	-6.19807800	0.22533500	2.16070300
O	-0.02624900	1.10886300	-4.05895300
H	0.48302300	1.40614600	-4.81810100
O	0.85763800	-0.40115300	-0.90197200
H	1.34610700	-1.08667600	-1.36628500
H	-1.04525200	0.90830100	-0.11509200
O	0.79477400	3.05997500	-1.91468500
C	0.95506800	2.44756200	0.47214800
H	0.29226000	2.19226200	1.30625000
H	1.68699900	1.64953100	0.35596600
O	1.60158700	3.68866500	0.71085700
H	2.19843700	3.57284100	1.45378000
O	-3.24918400	1.98539900	-0.03749700
O	-3.32856700	-0.62177600	-2.53849400
H	-3.50065300	-1.56677400	-2.49114200
O	-4.84871600	-1.76768600	-0.41588600
H	-5.74327700	-1.95260800	-0.11581300
C	-4.53407000	2.62250100	1.83752100
H	-5.47024500	2.42841600	2.36861600
H	-3.70291300	2.27103500	2.45870800
O	-4.40099100	4.00520600	1.55140500
H	-4.36718000	4.47904800	2.38587900
C	4.39390155	8.33366412	3.75176874
C	3.79036767	9.48664997	3.26601848
C	2.75155400	9.37626160	2.35233929
C	2.31508492	8.13069787	1.92514518
C	2.92520684	6.98403775	2.42015245
C	3.96770186	7.08289962	3.33401065
H	5.20541458	8.40541198	4.46547726
H	4.12643132	10.46069775	3.59711001
H	2.27157402	10.26656592	1.96492417
H	1.50330583	8.04187005	1.21211975
H	4.43293985	6.18010996	3.70991697
O	2.53342312	5.73194726	2.03922264
H	1.79630804	5.79667707	1.42393804

Table S16. Cellobiose -phenol-2 coordinates

C	-4.72188900	-0.36625300	-0.57506200
C	-3.37257500	-0.10629500	-1.22279200
C	-3.12499400	1.39840200	-1.31216800
C	-4.53913300	1.85133300	0.54180900
C	-4.85418400	0.36664300	0.74972400
H	-2.59589700	-0.56888500	-0.60269200
H	-5.50553700	0.00502900	-1.24825300

H	-5.29049700	2.28025400	-0.13511400
H	-4.13973400	-0.05421400	1.46530900
H	-3.85163100	1.84005200	-2.00370100
O	-1.87783400	1.73875500	-1.82650600
C	-0.73574900	1.34478300	-1.06477800
C	0.08449200	2.59665700	-0.76540100
C	0.04093300	0.27676200	-1.83907600
H	-0.62620300	3.39845200	-0.56007100
C	0.86709700	0.85895300	-2.98952100
H	-0.68665500	-0.41174900	-2.27625700
C	1.60471200	2.14487700	-2.61072500
H	1.61668300	0.11793600	-3.28444900
H	1.87335900	2.67885400	-3.52687500
O	2.76621000	1.79565600	-1.90049700
H	3.24149900	2.60682500	-1.69487400
O	-6.18212600	0.16882700	1.20234600
H	-6.19807800	0.22533500	2.16070300
O	-0.02624900	1.10886300	-4.05895300
H	0.48302300	1.40614600	-4.81810100
O	0.85763800	-0.40115300	-0.90197200
H	1.34610700	-1.08667600	-1.36628500
H	-1.04525200	0.90830100	-0.11509200
O	0.79477400	3.05997500	-1.91468500
C	0.95506800	2.44756200	0.47214800
H	0.29226000	2.19226200	1.30625000
H	1.68699900	1.64953100	0.35596600
O	1.60158700	3.68866500	0.71085700
H	2.19843700	3.57284100	1.45378000
O	-3.24918400	1.98539900	-0.03749700
O	-3.32856700	-0.62177600	-2.53849400
H	-3.50065300	-1.56677400	-2.49114200
O	-4.84871600	-1.76768600	-0.41588600
H	-5.74327700	-1.95260800	-0.11581300
C	-4.53407000	2.62250100	1.83752100
H	-5.47024500	2.42841600	2.36861600
H	-3.70291300	2.27103500	2.45870800
O	-4.40099100	4.00520600	1.55140500
H	-4.36718000	4.47904800	2.38587900
C	6.38046773	6.66164579	0.83267149
C	6.31506779	7.96586069	0.35903750
C	5.27603548	8.33039680	-0.48574317
C	4.30921936	7.40722700	-0.85648994
C	4.38391044	6.10572746	-0.37417201
C	5.42118240	5.72922056	0.47082254
H	7.18577690	6.36386726	1.49288820
H	7.06622305	8.69012899	0.64631901
H	5.21223256	9.34370158	-0.86291110
H	3.49620487	7.68978364	-1.51561824

H	5.46553380	4.71127617	0.83770576
O	3.45728758	5.15670671	-0.70137574
H	2.78510638	5.54358584	-1.27111314

Table S17. Cellobiose -phenol-3 coordinates

C	-4.72188900	-0.36625300	-0.57506200
C	-3.37257500	-0.10629500	-1.22279200
C	-3.12499400	1.39840200	-1.31216800
C	-4.53913300	1.85133300	0.54180900
C	-4.85418400	0.36664300	0.74972400
H	-2.59589700	-0.56888500	-0.60269200
H	-5.50553700	0.00502900	-1.24825300
H	-5.29049700	2.28025400	-0.13511400
H	-4.13973400	-0.05421400	1.46530900
H	-3.85163100	1.84005200	-2.00370100
O	-1.87783400	1.73875500	-1.82650600
C	-0.73574900	1.34478300	-1.06477800
C	0.08449200	2.59665700	-0.76540100
C	0.04093300	0.27676200	-1.83907600
H	-0.62620300	3.39845200	-0.56007100
C	0.86709700	0.85895300	-2.98952100
H	-0.68665500	-0.41174900	-2.27625700
C	1.60471200	2.14487700	-2.61072500
H	1.61668300	0.11793600	-3.28444900
H	1.87335900	2.67885400	-3.52687500
O	2.76621000	1.79565600	-1.90049700
H	3.24149900	2.60682500	-1.69487400
O	-6.18212600	0.16882700	1.20234600
H	-6.19807800	0.22533500	2.16070300
O	-0.02624900	1.10886300	-4.05895300
H	0.48302300	1.40614600	-4.81810100
O	0.85763800	-0.40115300	-0.90197200
H	1.34610700	-1.08667600	-1.36628500
H	-1.04525200	0.90830100	-0.11509200
O	0.79477400	3.05997500	-1.91468500
C	0.95506800	2.44756200	0.47214800
H	0.29226000	2.19226200	1.30625000
H	1.68699900	1.64953100	0.35596600
O	1.60158700	3.68866500	0.71085700
H	2.19843700	3.57284100	1.45378000
O	-3.24918400	1.98539900	-0.03749700
O	-3.32856700	-0.62177600	-2.53849400
H	-3.50065300	-1.56677400	-2.49114200
O	-4.84871600	-1.76768600	-0.41588600
H	-5.74327700	-1.95260800	-0.11581300
C	-4.53407000	2.62250100	1.83752100
H	-5.47024500	2.42841600	2.36861600

H	-3.70291300	2.27103500	2.45870800
O	-4.40099100	4.00520600	1.55140500
H	-4.36718000	4.47904800	2.38587900
C	3.17730437	1.07623008	2.55717335
C	2.97684148	-0.29765981	2.59988169
C	3.57538327	-1.10157310	1.63988156
C	4.36736743	-0.54551005	0.64590771
C	4.56258841	0.83036776	0.61493307
C	3.96641839	1.64513986	1.57014724
H	2.71641573	1.71519575	3.30039980
H	2.36082236	-0.73645633	3.37403124
H	3.42792005	-2.17439114	1.66059627
H	4.83660737	-1.17146257	-0.10461095
H	4.12791598	2.71530309	1.53206442
O	5.33637114	1.43186390	-0.33679969
H	5.70591837	0.76405559	-0.92294868
